# Comparative analysis of the chloroplast genomes of eight *Piper* species and insights into the utilization of structural variation in phylogenetic analysis

**DOI:** 10.3389/fgene.2022.925252

**Published:** 2022-09-29

**Authors:** Jing Li, Rui Fan, Jintao Xu, Lisong Hu, Fan Su, Chaoyun Hao

**Affiliations:** ^1^ Spice and Beverage Research Institute, Chinese Academy of Tropical Agricultural Sciences (CATAS), Wanning, Hainan, China; ^2^ Key Laboratory of Genetic Resources Utilization of Spice and Beverage Crops, Ministry of Agriculture and Rural Affairs, Wanning, Hainan, China; ^3^ Hainan Provincial Key Laboratory of Genetic Improvement and Quality Regulation for Tropical Spice and Beverage Crops, Wanning, Hainan, China; ^4^ Academician Soonliang Sim of Hainan Province Research Station, Wanning, Hainan, China; ^5^ Yangtze Normal University, Chongqing, China

**Keywords:** *piper*, chloroplast genome, phylogenetic relationships, DNA barcode, high degree of sequence variations

## Abstract

With more than 2000 species, *Piper* is regarded as having high medicinal, cosmetic, and edible value. There also remain some taxonomic and evolutionary uncertainties about the genus. This study performed chloroplast genome sequencing of eight poorly studied *Piper* species and a comparative analysis with black pepper (*Piper nigrum*). All examined species were highly similar in gene content, with 79 protein-coding genes, 24 tRNAs, and four rRNAs. They also harbored significant structural differences: The number of SSRs ranged from 63 to 87, over 10,000 SNPs were detected, and over 1,000 indels were found. The spatial distribution of structural differences was uneven, with the IR and LSC being relatively more conserved and the SSC region highly variable. Such structural variations of the chloroplast genome can help in evaluating the phylogenetic relationships between species, deciding some hard-to-distinguish evolutionary relationships, or eliminating improper markers. The SSC region may be evolving at high speed, and some species showed a high degree of sequence variation in the SSC region, which seriously affected marker sequence detection. Conversely, CDS sequences tended to lack variation, and some CDSs can serve as ideal markers for phylogenetic reconstruction. All told, this study provides an effective strategy for selecting chloroplast markers, analyzing difficult-to-distinguish phylogenetic relationships and avoiding the taxonomic errors caused by high degree of sequence variations.

## Introduction

The origin and evolution of plants have always been hot topics, especially the rapid evolution of angiosperms to become the dominant category of plants (Crane et al., 1995; Soltis and Soltis, 2004; Soltis et al., 2008; Magallón and Castillo, 2009; Sauquet and Magallón, 2018). Angiosperms have high diversity in morphology and traits, and their high degree of differentiation has made taxonomic classification challenging, leading to numerous taxonomic revisions over the years (Cai et al., 2006; Ruhlman et al., 2006; Hansen et al., 2007; Jansen et al., 2007; Berndt, 2013; Liu et al., 2020). *Piper* is a large genus of angiosperms with more than 2000 species, mainly distributed in the tropics. This genus occupies a special taxonomic position as a branch of the basal or sub-basal angiosperms (Group, 2016), and factors such as its origin, taxonomic position in angiosperms, unique traits, global distribution, and availability of high diversity in some areas makes *Piper* a genus of great scientific interest (Jaramillo et al., 2008; Asmarayani, 2018; Sen et al., 2019). Notably, the morphological features of plants can be greatly affected by environmental conditions, which makes it challenging to elucidate some aspects of taxonomy and evolution by means of classical methods that depend on leaf morphology, fruit morphology, and seed color (Tsoong Pu-Chiu, 1981; Ma, 1990; Liao et al., 2021). Chloroplasts are maternally-inherited organelles that have a conserved gene structure and undergo less gene recombination, which offers natural advantages in solving the problems of species classification and evolution (de Vries and Archibald, 2018; Nick, 2020). Thus, analysis of chloroplast genomes could be effective in determining the taxonomy of *Piper*, the mechanisms of species diversity, and the emergence and distribution of species.

Generally, chloroplast genomes vary in size from 107 to 218 kbp (Daniell et al., 2016), with *Piper* chloroplast genomes being about 160 kbp (Cai et al., 2006; Simmonds et al., 2021). The structure of the chloroplast genome is highly conserved, and can be divided into four regions, namely two inverted repeat (IR) regions separated by the large single copy (LSC) and small single copy (SSC) regions (Ruhlman and Jansen, 2014; Daniell et al., 2016). Although the chloroplast genome is highly conserved on the whole, the evolutionary rates of its different parts are distinct. The IR region is the most conserved while the SSC region is the fastest-changing; likewise, coding regions are conserved, and intronic and non-coding regions change rapidly. These characteristics make the chloroplast genome an important tool for undertaking accurate taxonomic classification (Cai et al., 2006; Daniell et al., 2016; Jin et al., 2019). That is, whole-chloroplast genome sequencing provides data support for the comprehensive analysis of different chloroplast regions and different types of sequence variations, and also provides the possibility for screening highly reliable and lineage-specific markers.

In phylogenetic analysis, barcodes or markers, including certain genes or certain fragments of genome sequences, are used to reconstruct relationships (de Vere et al., 2015; Liu et al., 2021). If markers have been developed for a certain group of species, the phylogenetic relationships of the group can be analyzed by direct sequencing of the designated markers. If no designated markers have been obtained for a group of plants, phylogenetic analysis may instead refer to a commonly-used marker that has not been verified. However, it can be difficult to fully elucidate the phylogenetic relationships between species when only considering common markers. Chloroplast markers or their sequences are often used to undertake large-scale phylogenetic analyses of species in a genus, and have made important contributions to high-precision classification in plant taxonomy. For example, chloroplast genomes have been used to compare the three orders of magnolias (Canellales, Magnoliales, and Piperales), with results strongly supporting the idea that these constitute a sister clade with monocots and eudicots (Cai et al., 2006). Regarding the genus *Piper*, the ITS sequences of 331 species and 181 accessions of psbJ-petA have been used to reconstruct the phylogenetic relationships of tropical species (Jaramillo et al., 2008). In addition, comparative genome analyses have been widely carried out in less-studied plants, improving our understanding of some rare and valued medicinal plants and giving support for the determination of evolutionary markers and species classification (Gao et al., 2018; Alwadani et al., 2019; Song et al., 2019; Favre et al., 2020).

Although the chloroplast genome follows maternal inheritance, a large number of studies have shown sequences to be widely distinct within a family or genus (Daniell et al., 2016; Song et al., 2019). Small-scale or local mutation events drive the chloroplast genome to change constantly; however, it is not yet confirmed whether overall structure variations of chloroplast genomes are correlated with the evolution of species, a question whose solving requires high-quality chloroplast genome assemblies. With the development of sequencing technology, the cost of chloroplast genome sequencing has decreased significantly, and new assembly and annotation tools have additionally made analysis of chloroplast genomes more convenient and reliable. Recent studies have indicated that the chloroplast genome can be used to solve the phylogenetic classifications of some closely related species (Cai et al., 2006; Song et al., 2019; Simmonds et al., 2021). Therefore, it can be expected that whole-chloroplast genome sequencing and more in-depth structure analysis will provide useful information for answering difficult and controversial evolutionary problems.

Herein, eight chloroplast genomes of *Piper* species were assembled with comparison to several published sequences. Then, *Piper nigrum* was introduced as a control and comparative analysis performed on it and the eight new assemblies. SSRs, tandem repeats, SNPs, and indels were analyzed using commonly-used specialist software. The spatial distribution of variations in *Piper* chloroplast genomes was investigated with sliding window analysis, and markers were screened according to their Pi values. Marker evaluation and phylogenetic analysis were combined with the comparative analysis of genome structural characteristics. Finally, marker screening based on protein-coding genes was carried out, and a marker screening strategy was proposed to overcome the abnormal variations observed in some *Piper* species.

## Materials and methods

### Plant materials, DNA extraction, and sequencing

A total of eight *Piper* species were selected for our study: *Piper boehmeriifolium*, *Piper austrosinese*, *Piper mutabile*, *Piper bonii*, *Piper betle*, *Piper retrofractum*, *Piper hainanense*, and *Piper umbellatum*. The plants were cultivated in the greenhouse of the Spice and Beverage Research Institute (Wanning, Hainan, China), and all samples were taken from fresh healthy leaves. A modified CTAB method was used to extract total genomic DNA (Pahlich and Gerlitz, 1980; Yang et al., 2014), the quality of which was assessed by spectrophotometer and agar-gel electrophoresis. DNA purity and concentration were surveyed using a nanospectrophotometer (OD values of qualifying samples were between 1.8 and 2.0), and then quantified using the Qubit2.0 fluorometer. The extracted chloroplast genome (1.0 µg) was next cut into 350 bp fragments by a CovarisS220 and short insert libraries constructed according to the procedures outlined in the Illumina manual. Subsequently, pair-end sequencing was performed using the Illumina Hiseq X platform.

### Assembly and annotations

NGSQCT was used to obtain high-quality sequencing data comprised of adaptor-free reads; the cut-off value for the percentage of read length was 80, and that for PHRED quality score was 30 (Patel and Jain, 2012). The plastomes of the eight *Piper* spices were then assembled using GetOrganelle (Jin et al., 2020). When annotating plastomes, six well-annotated species were used as references (*Piper auritum*, *Piper cenocladum*, *Piper kadsura*, *Piper laetispicum*, *Piper longum*, and *P. nigrum*). Six reference chloroplast genomes from *Piper* species were retrieved from NCBI (https://www.ncbi.nlm.nih.gov/), with accession number NC_034697.1 for *P. auritum*, NC_008457.1 for *P. cenocladum*, NC_027941.1 for *P. kadsura*, NC_042254.1 for *P. laetispicum*, NC_047247.1 for *P. longum*, and NC_034692.1 for *P. nigrum*. The software GeSeq and CPGAVAS2 were used to make the plastome annotations (Tillich et al., 2017; Shi et al., 2019). After annotation, a manual check was undertaken and the reading frames verified in Geneious by visual inspection of start and stop codons (Kearse et al., 2012). Transfer RNAs (tRNAs) were predicted using tRNAscan-SE 2.07 (Lowe and Eddy, 1997).

### Simple sequence repeat analysis

The microsatellite identification tool MISA was used to detect simple sequence repeats in six chloroplast genome sequences of *Piper* species (Beier et al., 2017), and each repeat sequence length was screened to be ≥10 bp. The minimum numbers of repeats required for mononucleotides, dinucleotides, trinucleotides, tetranucleotides, pentanucleotide, and hexanucleotides were 10, 5, 4, 3, 3, and 3, respectively. In addition, SSRs of the IR, LSC, and SSC regions were analyzed. Tandem repeat sequences were identified with TRF (Benson, 1999), with parameters of 2, 7, and 7 for matches, mismatches, and indels, respectively. Meanwhile, forward and palindromic repeats were identified using REPuter v1.0 (Kurtz et al., 2001) with the parameters of a minimal repeat size of 30 bp, hamming distance of 3 kp, and 90% sequence identity.

### Variation and comparison analyses of chloroplast genome sequences

All sequenced genomes were aligned using MAFFT and manually adjusted using Se-AI v2.0 (Rambaut, 1996; Katoh and Standley, 2013). In addition, SNPs and the genome microstructure were checked. SNPs were identified using MEGA 11.0 (Tamura et al., 2021), and indels were predicted using DnaSNP v6.0 (Rozas et al., 2017). A sliding window analysis was conducted to compare Pi values among the sequenced chloroplast genomes in DnaSNP v6.0, with window length 600 bp and step size 200 bp. The R package *ggcorrplot* was used to visualize the correlations between species (Kassambara, 2019).

### Codon usage analysis

RSCU, GC3s, and ENc values for protein-coding genes were calculated using CodonW v1.4.4 (Peden, 1999). Then, the relationship of ENc with GC3s was analyzed. The R package *ggplot2* was used to draw the ENC-GC3 plot (Wickham, 2016).

### Phylogenetic analysis

To investigate phylogenetic relationships, we used as outgroups a species from *Peperomia* (*Peperomia maculosa*) and a species from *Saururus* (*Saururus chinensis*). Sequence alignment was first carried out using MAFFT and then manually adjusted with Se-AI 2.0 (Rambaut, 1996). Phylogenetic analyses then were conducted using RAxML and MrBayes (Ronquist et al., 2012; Stamatakis, 2014), and phylogenetic trees reconstructed using the maximum likelihood (ML) and Baysian inference (BI) methods.

## Results

### Features of *piper* chloroplast genomes

**FIGURE 1:**
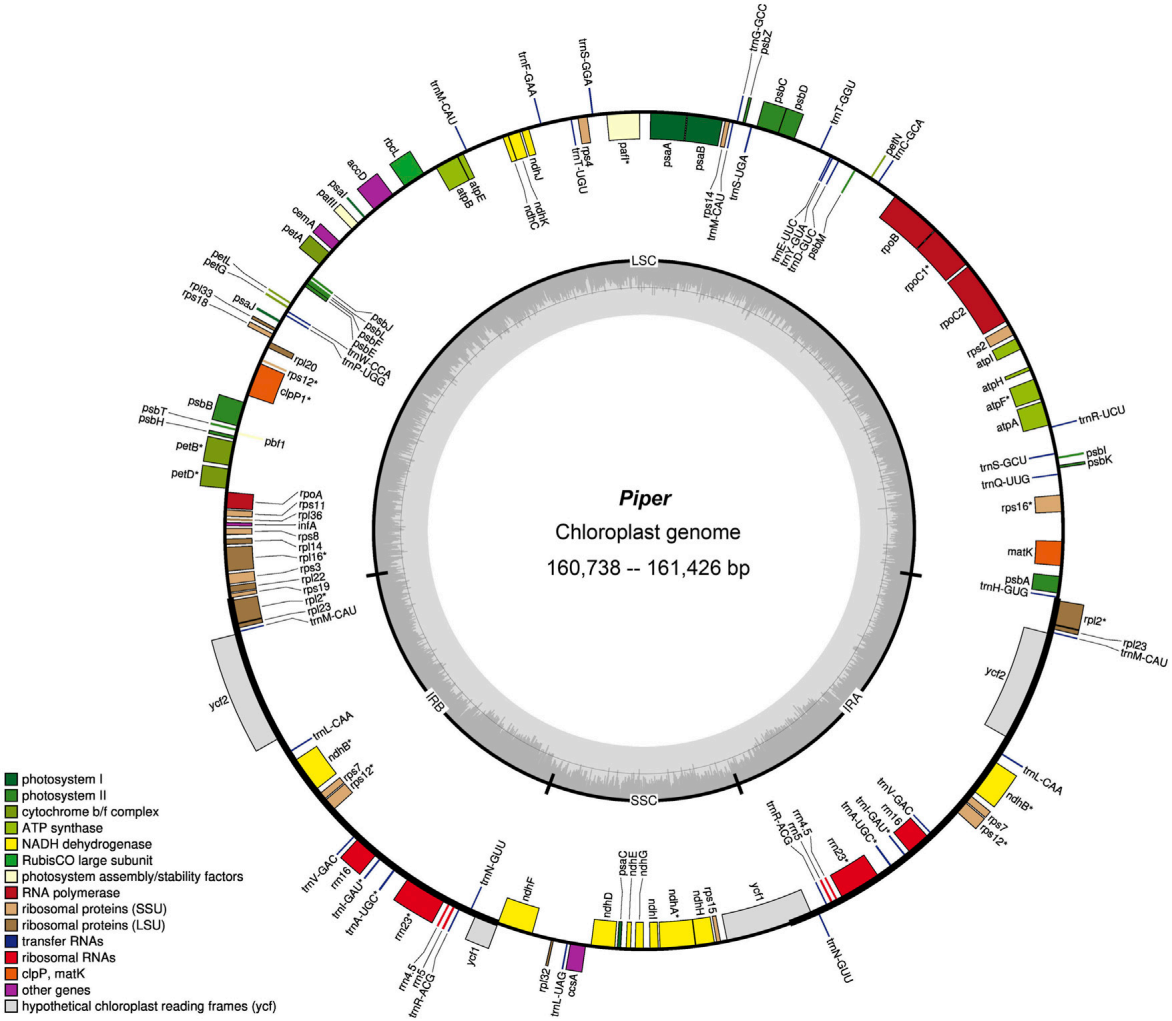
Gene map of *Piper* chloroplast genome. Genes belonging to different functional categories are color-coded. Genes inside the circle are transcribed in a clockwise direction and those located on the outside are transcribed in a counter-clockwise direction. LSC, large-single-copy; SSC, small-single-copy; IR, inverted repeat.

Overall, no significant differences were found in the eight assembled chloroplast genomes: Gene contents were similar, gene order was identical, and the three genomic regions were of similar sizes (LSC, 88,411–89,028 bp; SSC, 18,206–18,275 bp; and IR, 54,030–54,176 bp) (Figure 1 and Supplementary Figure S1). The GC content of the eight chloroplast genomes was 38.2%–38.4%, which is consistent with other reported *Piper* species (Simmonds et al., 2021). Detailed information on each chloroplast genome is recorded in Supplementary Table S1.

Regarding total genes, six species had 128 genes (*P. boehmeriifolium*, *P. austrosinese*, *P. mutabile*, *P. bonii*, *P. betle*, and *P. hainanense*), while there were 127 genes in both *P. retrofractum* and *P. austrosinense*. The number of protein-coding genes was highly similar across species, in that all contain 79 unique genes, and if duplicated genes are considered, six species contain 85 genes (*P. boehmeriifolium, P. mutabile, P. bonii, P. betle, P. hainanense,* and *P. umbellatum*) and two species contain 84 (*P. austrosinense* and *P. retrofractum*). The counts of tRNAs and rRNAs were the same in all eight assembled genomes (33 tRNAs and 10 rRNAs). Notably, six protein-coding genes (*ndhB*, *rpl2*, *rpl23*, *rps7*, *ycf1*, and *ycf2*), seven tRNA genes (trnA-UGC, trnI-GAU, trnL-CAA, trnM-CAU, trnN-GUU, trnR-ACG, trnV-GAC), and all four rRNA genes were duplicated in the IR regions. All *Piper* chloroplast genomes harbored 14 intron-containing genes, of which ten protein-coding genes (*rps16*, *atpF*, *rpoC*, *petB*, *petD*, *rpl16*, *rpl2*, *ndhB*, *ndhA*, and *rps12*) and two tRNA genes had a single intron, and two genes (*pafl* and *clpP*) had two introns. Of particular note is *rps12*, a trans-splicing gene, with the 5′ end located in the LSC region and the duplicated 3′ end in the IR region.

### Repeat sequence analyses

**FIGURE 2:**
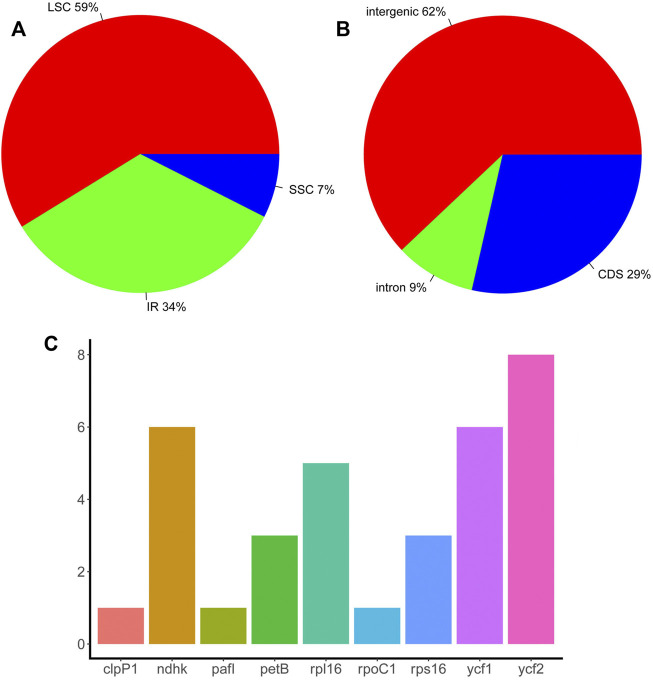
The distribution of repeat sequences in *Piper* chloroplast genomes. **(A)** The proportion of repeat sets in the three regions (IR, LSC, and SSC). **(B)** The proportion of repeat sets in introns, intergenic regions, and CDS. **(C)** The number of repeat sets in different genes.

Repeat sequences play an important role in chloroplast genome evolution and affect genome rearrangement and recombination. Here, TRF was used to predict repeat sequences in the chloroplast genomes of eight *Piper* species using two separate thresholds, 100% match and >90% match. When requiring 100% match, 18 sets of repeats were identified in *P. boehmeriifolium*, 17 in *P. austrosinese*, 23 in *P. mutabile*, 19 in *P. bonii*, 22 in *P. betle*, 18 in *P. retrofractum*, 22 in *P. hainanense*, and 10 in *P. umbellatum*. With the >90% match criterion, another 11 were identified in *P. boehmeriifolium*, 5 in *P. austrosinese*, 12 in *P. mutabile*, 8 in *P. bonii*, 8 in *P. betle*, 10 in *P. retrofractum*, 11 in *P. hainanense*, and 9 in *P. umbellatum*. In general, the number of repeat regions varied greatly among *Piper* species, as did the locations and numbers of repeats, with *P. umbellatum* showing the most significant difference. Overall, 133 sets of repeats were identified in the LSC, 17 sets in the SSC, and 73 sets in the IR (Figure 2A). Repeat sequences were distributed among gene regions, with 29% in CDSs, 9% in introns, and 62% in intergenic regions (Figure 2B). With respect to genes, nine featured repeats in their CDS or intron regions, including *rpl16*, *ycf2*, *ndhk*, *ckpP1*, *ycf1*, *petB*, *rps16*, *pafI*, and *rpoC1*. Of those, four were found to have repeats in more than half of the investigated species, and *ycf2* harbored repeats in all species (Figure 2C).

**FIGURE 3:**
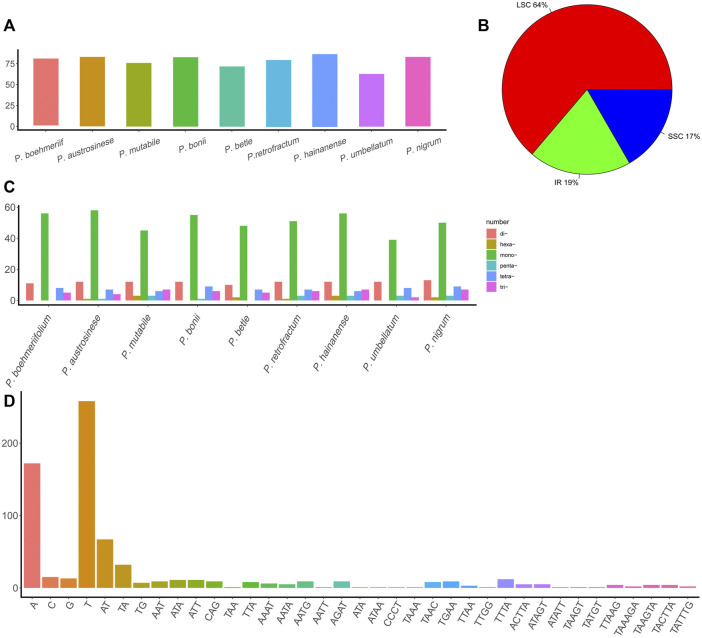
The SSRs in *Piper* chloroplast genomes. **(A)** The number of SSRs in different *Piper* species. **(B)** The proportion of SSRs in the three regions (IR, LSC, and SSC). **(C)** The number of different types of SSRs (mononucleotides, dinucleotides, trinucleotides, tetranucleotides, pentanucleotides, and hexanucleotides). **(D)** The number of each SSR.

This study also examined the occurrence of SSRs in chloroplast genomes (Supplementary Table S2). Total SSR numbers in each species ranged from 63 to 87 (Figure 3A). Most SSRs were found in the LSC (64%), and the remainder about equally distributed between the IR (19%) and SSC (17%) (Figure 3B). Except for *P. umbellatum*, all species harbored more than 70 SSRs, and five species had more than 80 SSRs. Of the identified SSRs, 39–56 were mononucleotides, 10–13 were dinucleotides, 2–7 were trinucleotides, 4–9 were tetranucleotides, and 1–3 were pentanucleotides and hexanucleotides (not all species had each type of SSR) (Figure 3C). A total of 37 types of SSRs were identified (Figure 3D), and A and T nucleotides were present in extremely high proportions among mononucleotide and polynucleotide SSRs.

### SNP analyses

**FIGURE 4:**
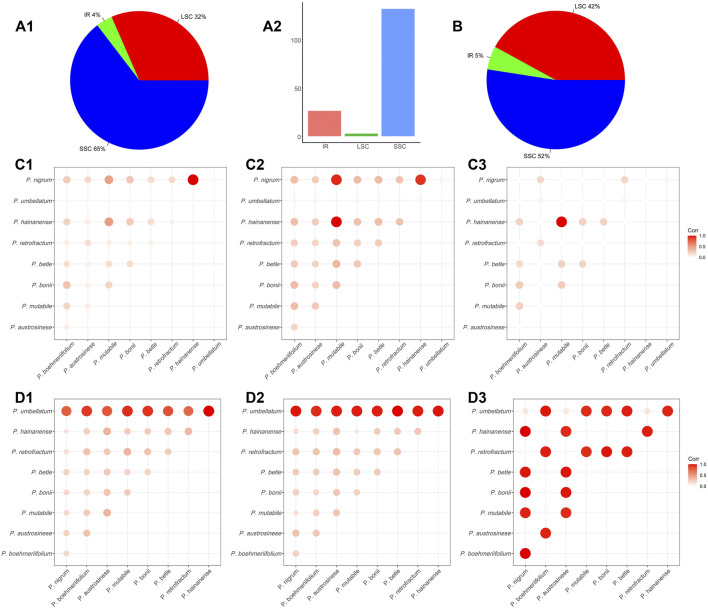
The SNP and indel in *Piper* chloroplast genomes. **(A)** The **(A1)** shows the proportion of SNP in the three regions (IR, LSC, and SSC). The **(A2)** shows the average number of nucleic acids per SNP in the three regions (IR, LSC, and SSC). **(B)** The proportion of indels in the three regions (IR, LSC, SSC). **(C)** The heat map of the correlations between species that constructed by the average length of the nucleic acid sequence in which an SNP appears. Each value was normalized by dividing the maximum value. A higher value indicates a greater correlation between the two species. **(D)** The heat map of correlations between species that constructed by the number of indels. Each value was normalized by dividing the maximum value. A higher value indicates a lower correlation between the two species.

The abundance of SNPs and indels in a genome reflects its degree of conservation to a certain extent; accordingly, we also investigated the presence of these two types of sequence variation in chloroplast genomes. Regarding SNPs, a total of 10,744 SNPs were detected in the nine *Piper* chloroplast genomes. The numbers of SNPs in each of the three genome regions differed greatly, with 3,392 (32%) in the LSC, 411 (4%) in the IR, and 6,941 (65%) in the SSC (Figure 4A1). As shown in Figure 4A2, the IR region featured one SNP per 131.7 nucleic acids on average, and constituted the most stable region. The other two regions were comparatively less conserved, with one SNP per 26.2 nucleic acids in the LSC and one SNP per 2.6 nucleic acids in the SSC (Figure 4A2). The SSC region exhibited the highest SNP variation rate, indicating that this region is in high speed of evolving in *Piper* species.

Overall, the total number of SNPs in the investigated species was too large to be readily analyzed, so to more clearly elucidate the sequence variations between species, SNP content was analyzed in a pairwise manner. To compare species, the SNPs in each of the three regions (LSC, IR, SSC) were tabulated, and the average length of the nucleic acid sequence in which a SNP appears (ALS) was also evaluated. A larger ALS value indicates less difference in SNP number between the two species, and suggests the two sequences may be more similar. In this way, we analyzed the sequence similarity of each genomic regions for each pair of species. For clarity, we visualized similarity using heat maps and normalized each pairwise ALS value by dividing it by the maximum ALS, guaranteeing a normalized value of between 0 and 1. In general, the LSC and IR regions showed high similarity in ALS values and hence are highly similar (Figures 4C1,C2). However, an obviously different pattern was exhibited by the SSC (Figure 4C3), with values ranging from 165 to 4,474 (Supplementary Table S3). As shown in Figures 4C1 and C2, the largest ALS values were obtained in comparisons involving *P. mutabile*, *P. hainanense*, and *P. nigrum*, and the smallest in comparisons with *P. umbellatum*.

In addition, nucleotide substitution was also plotted in a bar chart in Supplementary Figure S2. There were 5,810 transitions (Ts) and 5,470 transversions (Tv) and the Ts to Tv ratio was 1.06, indicating a bias in favor of transitions. C to T and G to A are the most frequently occurring mutations (3,082) while C to G and G to C exhibited the lowest frequency (608).

### Indel analyses

We then assessed the indels in the nine species. Across all species combined, a total of 1,056 indels were found. In terms of genomic distribution, indels were similar to SNPs, with 444 in the LSC (42%), 58 in the IR (5%), and 554 in the SSC (52%) (Figure 4B). We also conducted pairwise comparisons of the numbers of indels in the investigated species (Supplementary Table S4) and found them to vary considerably, ranging from 18 to 508. There were very obviously more indels in *P. umbellatum* compared to the other species, and this trend was evident in both the IR and LSC. The correlations between species were visualized with a heat map. Figures 4D1–D3 depicts such a comparison, with bubble size reflecting the number of indels; specifically, a larger bubble indicates less sequence consistency between the corresponding two species. In the heat maps for the LSC and IR, it is obvious that for all species, the greatest number of indels occurred in comparisons involving *P. umbellatum*, and the two regions were generally similar in overall pattern.

### Preliminary screening of genomic markers

**FIGURE 5:**
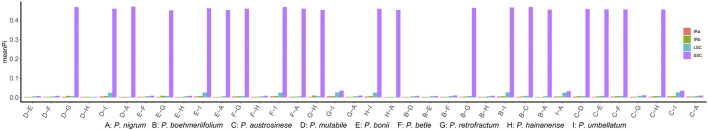
The mean Pi values between species.

To elucidate the composition and sequence variation of chloroplast genomes, a sliding window analysis was performed and sequence divergence degree determined in terms of the variation in Pi values (Supplementary Table S5). The SNP and indel analyses indicated that the sequence variation in some regions may be quite extensive; accordingly, we analyzed each of the three regions separately. Firstly, we performed a sliding window analysis on each pair of species. Figure 5 shows the mean Pi value obtained for each species pair in each region. Overall, the IR region exhibited quite low mean Pi values, which is in sharp contrast to the SSC region; 20 pairs had a mean Pi of more than 0.4 in the SSC region, indicating a high degree of sequence variation. Divergence in the LSC region was generally a little greater than in the IR and quite lower than in the SSC.

**FIGURE 6:**
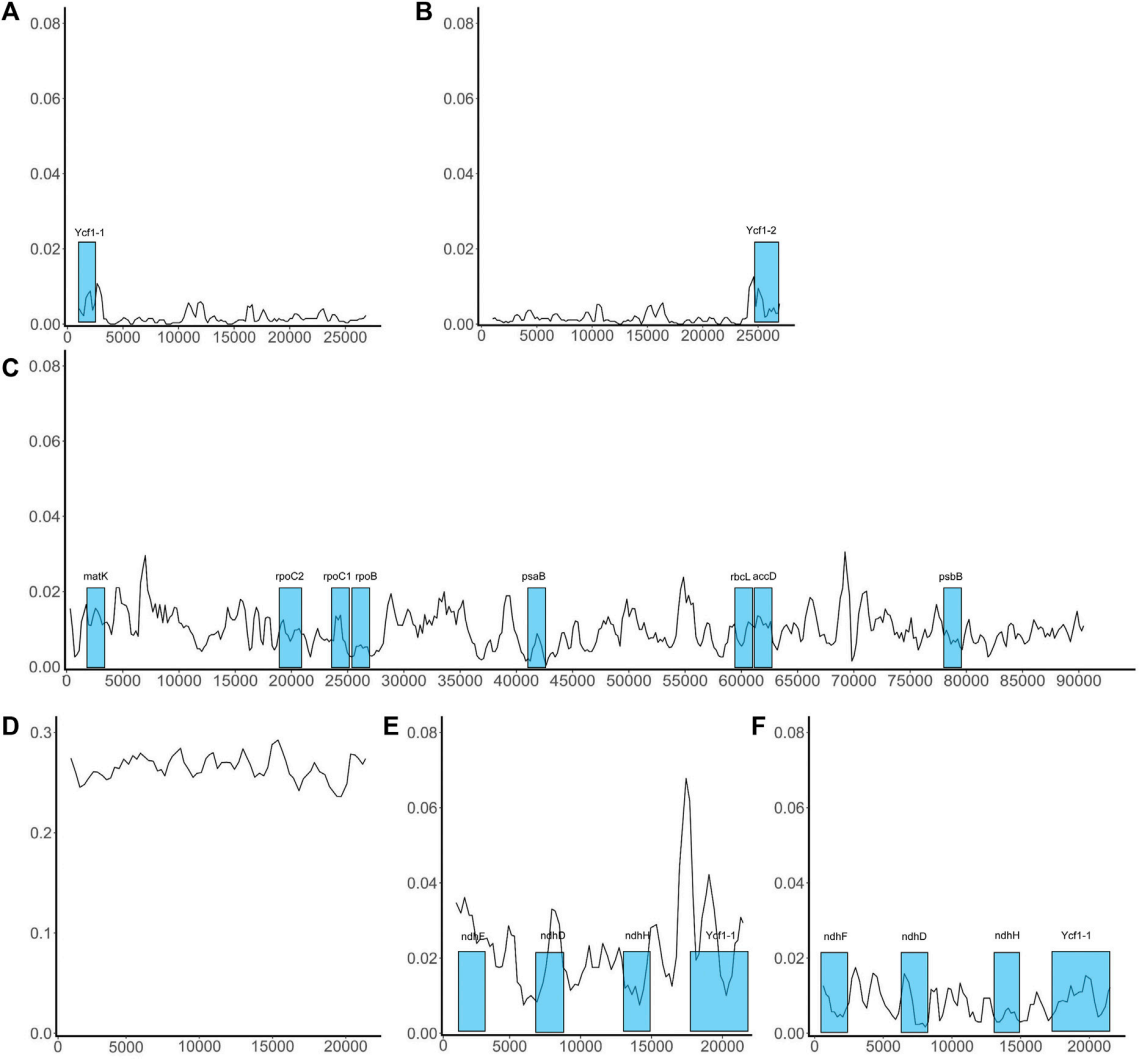
Sliding window analysis of the whole chloroplast genome of *Piper* species. *X*-axis shows the position; *Y*-axis, nucleotide diversity of each window. The coding genes that can be used as barcodes were screened out and their locations are highlighted in blue boxes. **(A)** The distribution of Pi values in IRa region; **(B)** The distribution of Pi values in IRb region; **(C)** The distribution of Pi values in LSC region; **(D)** The distribution of Pi values in SSC region; **(E)** The distribution of Pi values in SSC region of the species set with 5 species; **(F)** The distribution of Pi values in SSC region of the species set with 4 species.

Variation in conserved sequences of the chloroplast genome can reflect the degree of species differentiation, can be used in screening barcodes or markers, and can be used to reconstruct phylogenetic relationships. However, we are concerned that the excessive differences identified in SSC regions may adversely affect barcode screening. We further grouped species based on sequence similarity in the SSC region: If two species had a relatively small mean Pi value, they were included in the same group. This method grouped the nine species into two sets, one comprising five species (*P. boehmeriifolium*, *P. mutabile*, *P. bonii*, *P. betle*, and *P. hainanense*) and the other four species (*P. austrosinese*, *P. retrofractum*, *P. umbellatum*, and *P. nigrum*). We then considered the sequence characteristics of the SSC both among the nine species and within the respective species sets. Figure 6 displays a sliding window analysis of the whole genome. As mentioned above, the IR region was the most stable, with all Pi values less than 0.02 (Figures 6A,B). In contrast, the LSC was unstable, with many Pi values above 0.02 (Figure 6C). Finally, the SSC was the most unstable region, with Pi values of more than 0.2 (Figure 6D). When examining each group separately, the degree of sequence variation within each set was much lower and close to the values of the IR and LSC regions (Figures 6E,F). This finding indicates that the differences among species within each set are small, but the variation between the two sets is significantly high.

### Structural analysis of region borders

**FIGURE 7:**
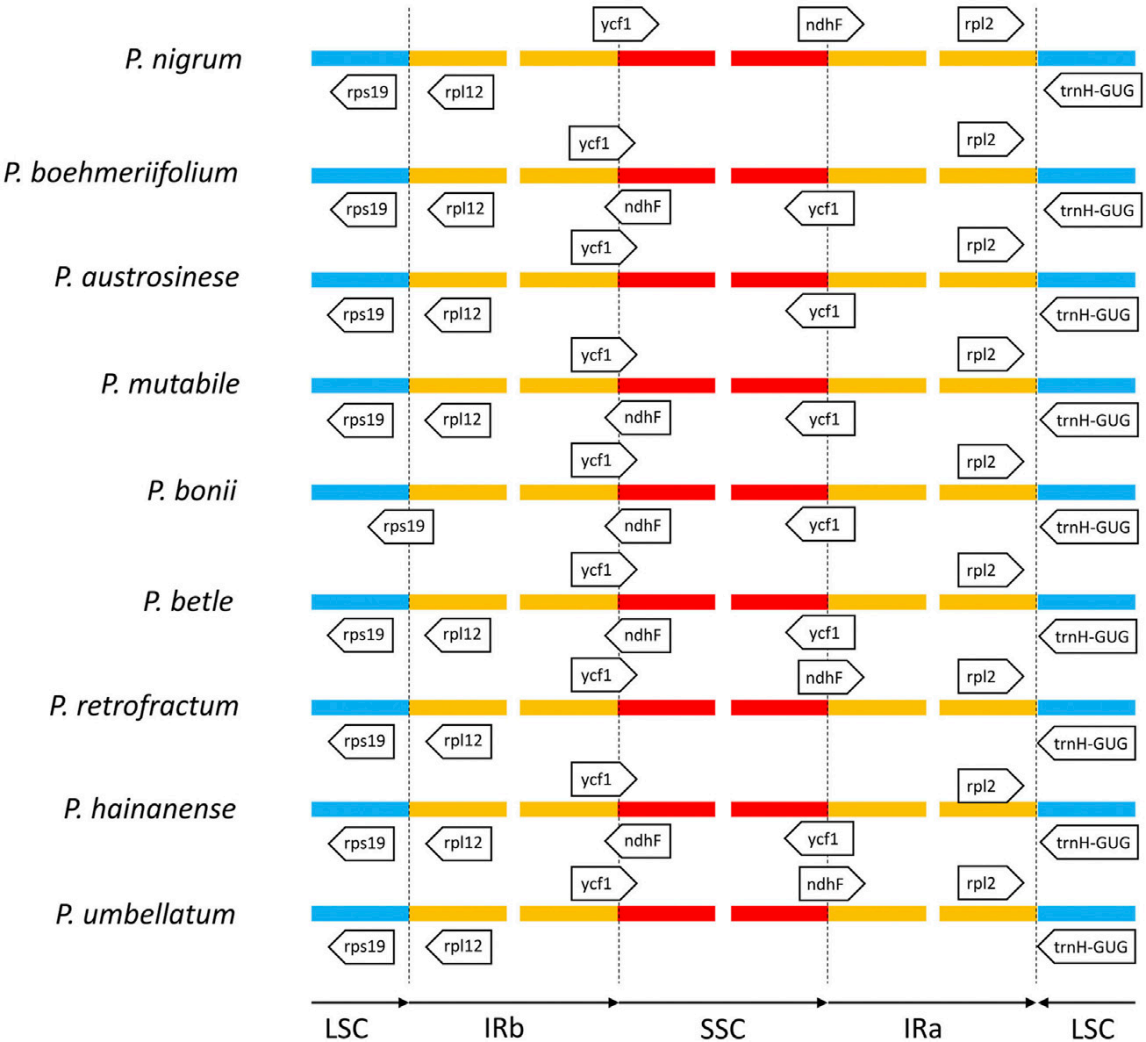
Comparison of boundaries among IR, LSC, and SSC regions of nine *Piper* species. Genes above lines are transcribed forward and those below the lines are transcribed reversely.

Next, we analyzed the boundaries of the three regions (Figure 7). The junction between the LSC and IRa was the least divergent due to the presence of a positive coding gene and a negative tRNA gene (trnH-GUG) on either side of the junction. The distances from the two genes to the boundary were typically only a few dozen to less than two hundred nucleic acids. We also found the boundary between the LSC and IRb to be relatively stable in species other than *P. bonii*, with two coding genes *rps19* and *rps12* distributed on either side. Notably, the boundary gene distribution of the SSC and IRb will influence that of the SSC and IRa. That is, if only the gene *ycf1* is located between the IRb and SSC, the boundary between the SSC and IRa features the positive-direction *ndhF* gene; conversely, if the two partially overlapping genes *ycf1* and *ndhF* are located span the boundary of the IRb and SSC, only *ycf1* would be located span the SSC and IRa boundary.

### Codon bias analysis

**FIGURE 8:**
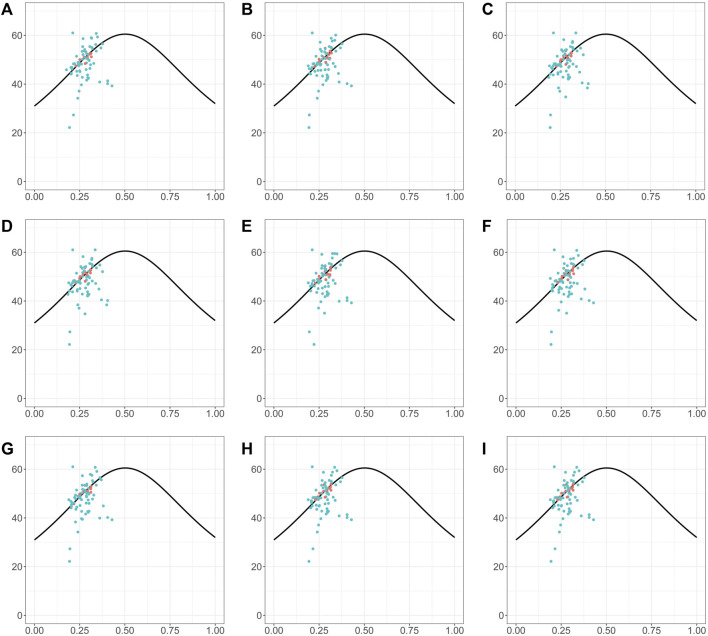
The ENc-GC3 plot of different *Piper* species. The solid line represents the expected curve of positions of genes when the codon usage was only determined by the GC3s composition. The red dots highlighted the screened markers. **(A)** *P. nigrum*; **(B)** *P. boehmeriifolium*; **(C)** *P. austrosinese*; **(D)** *P. mutabile*; **(E)** *P. bonii*; **(F)** *P. betle*; **(G)** *P. retrofractum*; **(H)** *P. hainanense*; **(I)** *P. umbellatum*.

The ENC-GC3 plot can indicate the specific selection preference of codons during gene evolution. ENC reflects the number of effective codons in a sequence of amino acids, and can have values between 20 and 61. The smaller the ENC value, the stronger the bias in codon use. In general, highly expressed genes have higher degree of codon preference, while lowly expressed genes may contain a greater diversity of rare codons and so have higher ENC values. In the species investigated here, the ENC-GC3 patterns were generally similar (Figure 8). More than half of genes were below the expected curve, indicating that they may have been affected by stronger selection pressures, that they may be more highly expressed, and also that natural selection exerted a strong influence on the codon bias of the chloroplast genome (Figure 8). In addition, there were also many genes with ENC values above the expected curve, suggesting that a relatively high proportion of genes in *Piper* chloroplasts are under loose selection pressure and may have lower expression levels (Figure 8).

### Phylogenetic reconstruction and protein-coding gene analysis

**FIGURE 9:**
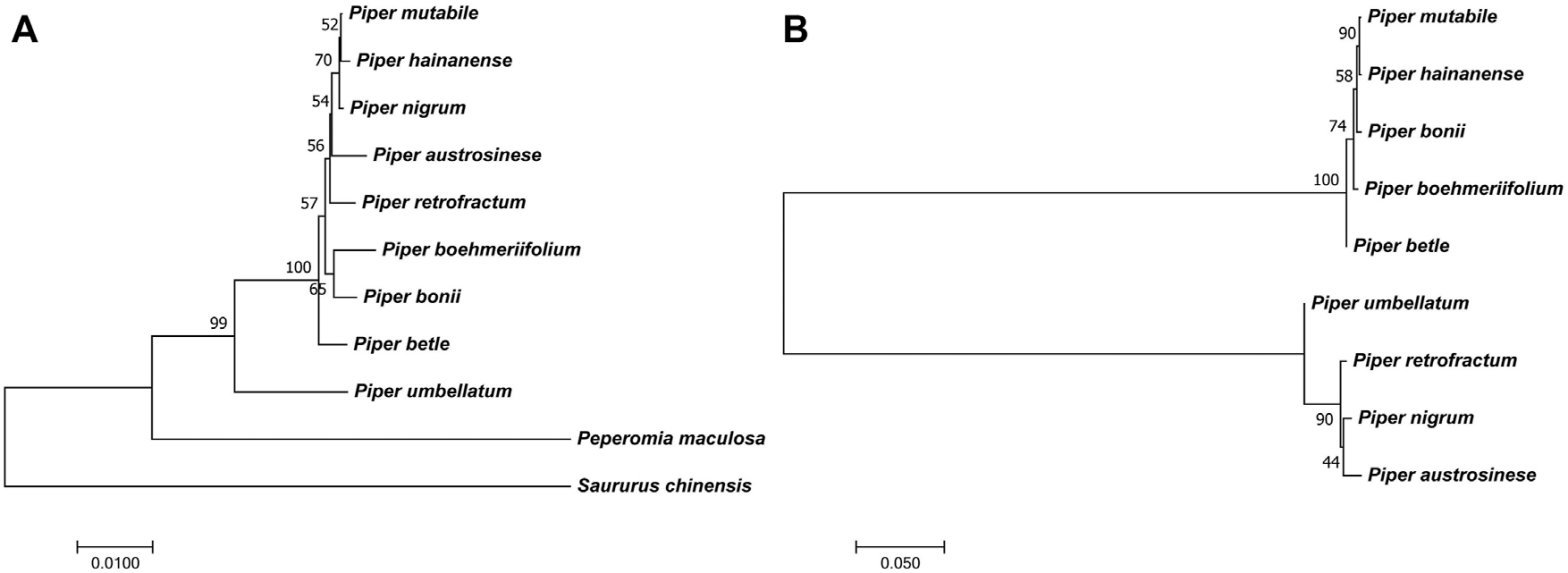
The phylogenetic trees. **(A)** The phylogenetic tree of *Piper* species [A tree constructed from screened markers. The other trees from the sequence and SNPs of IR and LSC also showed similar results (Supplementary Figure S3)]. **(B)** The conflicting phylogenetic relationships that constructed by unsuitable markers from SSC region [The trees from SNPs and sequence of SSC region also showed similar result (Supplementary Figure S3)].

To obtain accurate phylogenetic relationships, we performed phylogenetic analyses on highly variable sequence intervals and all coding sequences, illustrated in Figure 9. Then, we additionally performed a phylogenetic analysis for each coding gene, and identified 12 coding genes that can reflect the phylogenetic relationships of *Piper* species, namely *accD*, *matK*, *ndhD*, *ndhF*, *ndhH*, *psaB*, *psbB*, *rbcL*, *rpoB*, *rpoC1*, *rpoC2*, and *ycf1*. Based on the length and location of each gene, we located each on the genome as shown in Figure 6. Table 1 lists the corresponding sequence features, including CDS length after multiple sequence alignment, SNP number, SNP number per site, gene functional classification, and indel site number. All selected sequences are marked in red. We found that genes of more than 1,000 bp in length and harboring more than 30 SNPs tend to be ideal for reflecting the phylogenetic relationships among *Piper* species (henceforth referred to as “ideal genes”). We also observed that the average number of SNPs per site had no significant influence on phylogenetic relationship. Indels were found in very few genes, and indel number likewise had no significant influence on phylogenetic relationship. In terms of gene function, we found that NdH oxidoreductase genes (*ndh−*) and DNA-directed RNA polymerase subunit alpha genes (rpo*−*) tend to better reflect phylogenetic relationships. Ultimately, three rpo-genes (*rpoB*, *rpoC1*, and *rpoC2*), which account for 75% of all rpo- genes, met the criteria to be considered ideal genes.

**TABLE 1:** The properties of protein-coding genes.

Gene	CDS length (bp)	SNPs	SNP per site	Gene function classification	Indel sites
*atpA*	1491	33	0.022133	ATP synthase	0
*atpB*	1467	30	0.02045	ATP synthase	0
*atpE*	388	14	0.036082	ATP synthase	0
*atpF*	541	14	0.025878	ATP synthase	0
*atpH*	239	7	0.029289	ATP synthase	0
*atpI*	729	15	0.020576	ATP synthase	0
*ndhA*	1061	31	0.029218	NadH oxidoreductase	0
*ndhB*	1530	3	0.001961	NadH oxidoreductase	0
*ndhC*	354	9	0.025424	NadH oxidoreductase	0
*ndhD*	1453	44	0.030282	NadH oxidoreductase	0
*ndhE*	300	6	0.02	NadH oxidoreductase	0
*ndhF*	2106	117	0.055556	NadH oxidoreductase	3
*ndhG*	514	17	0.033074	NadH oxidoreductase	0
*ndhH*	1153	29	0.025152	NadH oxidoreductase	0
*ndhI*	523	20	0.038241	NadH oxidoreductase	0
*ndhJ*	463	14	0.030238	NadH oxidoreductase	0
*ndhK*	672	18	0.026786	NadH oxidoreductase	72
*petA*	944	19	0.020127	cytochrome b6/f complex	0
*petB*	637	11	0.017268	cytochrome b6/f complex	0
*petD*	474	9	0.018987	cytochrome b6/f complex	21
*petG*	114	0	0	cytochrome b6/f complex	0
*petL*	95	1	0.010526	cytochrome b6/f complex	0
*petN*	87	3	0.034483	cytochrome b6/f complex	0
*psaA*	2209	44	0.019919	Photosystem I	0
*psaB*	2168	37	0.017066	Photosystem I	0
*psaC*	237	9	0.037975	Photosystem I	0
*psaI*	109	2	0.018349	Photosystem I	0
*psaJ*	134	1	0.007463	Photosystem I	0
*psbA*	1046	16	0.015296	Photosystem II	0
*psbB*	1491	36	0.024145	Photosystem II	0
*psbC*	1361	25	0.018369	Photosystem II	36
*psbD*	1045	20	0.019139	Photosystem II	0
*psbE*	251	1	0.003984	Photosystem II	0
*psbF*	119	1	0.008403	Photosystem II	0
*psbH*	218	4	0.018349	Photosystem II	0
*psbI*	107	4	0.037383	Photosystem II	0
*psbJ*	122	1	0.008197	Photosystem II	0
*psbK*	177	3	0.016949	Photosystem II	0
*psbL*	116	1	0.008621	Photosystem II	0
*psbM*	105	0	0	Photosystem II	0
*psbT*	105	3	0.028571	Photosystem II	0
*psbZ*	187	2	0.010695	Photosystem II	0
*rpl2*	818	4	0.00489	Ribosomal Proein	0
*rpl14*	364	5	0.013736	Ribosomal Proein	0
*rpl16*	408	13	0.031863	Ribosomal Proein	0
*rpl20*	371	13	0.03504	Ribosomal Proein	3
*rpl22*	406	17	0.041872	Ribosomal Proein	9
*rpl23*	282	0	0	Ribosomal Proein	0
*rpl32*	163	2	0.01227	Ribosomal Proein	0
*rpl33*	202	5	0.024752	Ribosomal Proein	0
*rpl36*	111	3	0.027027	Ribosomal Proein	0
*rpoA*	985	35	0.035533	DNA-directed RNA polymerase subunit alpha	9
*rpoB*	3153	60	0.019029	DNA-directed RNA polymerase subunit alpha	0
*rpoC1*	1994	58	0.029087	DNA-directed RNA polymerase subunit alpha	15
*rpoC2*	3993	132	0.033058	DNA-directed RNA polymerase subunit alpha	18
*rps2*	692	19	0.027457	Ribosomal Protein	0
*rps3*	639	24	0.037559	Ribosomal Protein	0
*rps4*	591	15	0.025381	Ribosomal Protein	0
*rps7*	466	2	0.004292	Ribosomal Protein	0
*rps8*	391	14	0.035806	Ribosomal Protein	0
*rps11*	409	8	0.01956	Ribosomal Protein	0
*rps12*	371	1	0.002695	Ribosomal Protein	0
*rps14*	298	5	0.016779	Ribosomal Protein	0
*rps15*	265	8	0.030189	Ribosomal Protein	0
*rps16*	285	9	0.031579	Ribosomal Protein	0
*rps18*	306	3	0.009804	Ribosomal Protein	0
*rps19*	264	15	0.056818	Ribosomal Protein	0
*accD*	1491	47	0.031522	others	51
*ccsA*	930	36	0.03871	others	0
*cemA*	668	22	0.032934	others	0
*clpP1*	602	7	0.011628	others	0
*infA*	226	8	0.035398	others	0
*matK*	1467	72	0.04908	others	12
*pafI*	499	8	0.016032	others	0
*pafII*	537	18	0.03352	others	0
*pbf1*	129	3	0.023256	others	0
*rbcL*	1373	55	0.040058	others	0
*ycf1*	5077	320	0.063029	others	93
*ycf2*	6886	47	0.006825	others	24

The phylogenetic relationships of the investigated species are depicted in Figure 9A. To ensure the accuracy of the evolutionary relationship, markers, SNPs and the sequences of different regions were all used to reconstruct the phylogenetic trees (Supplementary Figure S3). Overall, the greatest evolutionary distance was identified between *P. umbellatum* and all other species, while the shortest evolutionary distances were found between the three species of *P. mutabile*, *P. hainanense*, and *P. nigrum*. However, when we analyzed sequences having significant variation in the SSC region, distinct evolutionary relationships were obtained (Figure 9B and Supplementary Figure S3). Notably, the tree clustered into two distinct branches having five and four species each, consistent with the Pi analysis. Interestingly, we also found that some protein-coding genes in the SSC are better able to reflect phylogenetic relationships. Four such genes were identified as ideal genes (Table 1 and Figure 6), which suggests their CDSs did not experience high rate of evolving.

## Discussion

*Piper* is a large genus of flowering plants, and its member species are diverse in morphological and physiological characteristics. There remain many problems in categorizing *Piper* species exactly, and also in elucidating the precise genetic and evolutionary processes underlying some key traits of the genus. New articles on *Piper* classification continue to be published, placing the evolutionary relationships of some species in a state of constant revision and update (Jaramillo et al., 2008; Frenzke et al., 2015; Asmarayani, 2018; Sen et al., 2019). Indeed, some questions about the emergence and spread of *Piper* species have puzzled researchers for a long time, for example why some regions feature many species within a narrow geographic area, such as the mountainous areas of Yunnan in China. Reconstructing an accurate phylogenetic tree is a key step in understanding the generation and diffusion of a series of new species. Phylogenetic relationships inferred from the chloroplast genome have been demonstrated able to solve taxonomic problems involving highly diverse and taxonomically challenging groups such as Piperaceae (Huang et al., 2019; Simmonds et al., 2021). It has also been proven that compared with utilizing only a small portion of sequence or a common barcode, analyzing the whole plastome allows phylogenetic relationships to be resolved with high reliability and support (Daniell et al., 2016; Simmonds et al., 2021). Moreover, whole-chloroplast genome screening provides the opportunity to identify the most suitable linkage-specific sequence markers. In this study, we generated chloroplast genome assemblies for eight *Piper* species and carried out a comprehensive comparative analysis. By analyzing overall genome structure and screening the highly variable regions, we identified ideal markers that are informative for the phylogenetic relationships of *Piper* species.

This study mainly focuses on how to solve the issues that hinder phylogenetic analyses based on chloroplast genomes, such as marker sequence screening, excluding the influence of abnormally variable genomic regions, and integrating multiple sequence variation characteristics to assist in evaluating the phylogenetic relationships between species. All species here showed obvious sequence differences and considerable phylogenetic distance from *P. umbellatum*. Notably, structural differences in chloroplast genomes may relate to significant phenotypic differences and long geographical isolation (Lim et al., 2019; Sen et al., 2019). As such, we believe that the purpose of genome comparisons should not merely be limited to simple prediction of structural variations among species but should also consider the associations of structural variations with the evolutionary distance between species. These correlations are likely to be an important means of elucidating the phylogenetic relationships between species.

Some genomic features can assist in determining phylogenetic relationships. Here, we explored the overall characteristics of chloroplast genomes in different species through pairwise comparative analysis. Generally, the IR region exhibits the least sequence variation and is the most stable region; this aligns with the relatively high variation of LSC and SSC regions reported in previous studies (Gao et al., 2018; Song et al., 2019; Fan et al., 2021; Simmonds et al., 2021). A previous study also showed the SSC region of *Piper* to present a relatively higher average Pi value fluctuation (Simmonds et al., 2021), though the values obtained were not as high as in our study. Herein, if all species are considered together, the SSC region showed high degree of differention; however, when the species are clustered into two groups, the differences within each set were significantly reduced and the obtained Pi values were highly consistent with the previous study. Thus, species may differ in the degree of sequence variation within the SSC, and such variation may not be shared by closely related species. This leads to the identification of many high-Pi-value sequences that cannot correctly reflect the phylogenetic relationship. For example, *P. nigrum*, *P. mutabile*, and *P. hainanenseare* are evolutionarily close, but did not all group into the same set (Figure 9B); meanwhile, *P. umbellatum* is evolutionarily distant from all other examined species, but clustered into a set with three other species (Figure 9B). Due to the uniqueness of sequence variation, it is not appropriate to directly use Pi values when screening marker sequences in the SSC; doing so usually leads to incorrect phylogenetic reconstruction, resulting in erroneous separation of species sets and an inability to reconstruct the real evolutionary relationships. Compared with the SSC region, the LSC and IR regions are more suitable for determining markers directly according to Pi value.

SNPs, SSRs, and indels are several important characteristics commonly used in assessing chloroplast sequence variation (Marie-France et al., 2004; Gaudeul et al., 2011; Jiao et al., 2012; Piya and Nepal, 2013). We found that some characteristics can effectively assist species classification and phylogenetic reconstruction when conducting a comprehensive pair-wise comparison. The number and location of SSRs can reflect the evolutionary distance between species to a certain extent. For example, it can be seen that *P. umbellatum* is very different from the other investigated species, having a much lower SSR count (63 vs. > 70). In addition, we also observed a large number of SSRs in similar locations among species that are close in evolutionary distance (Supplementary Table S2). SNPs can also be used to generate preliminary estimates of the evolutionary distance between species. Here, for all nine species combined, a total of over 10,000 SNPs was obtained. It seems that the *Piper* chloroplast genome is highly unstable and the SNPs densely distributed between species. However, pairwise comparisons of the SNP distributions between species revealed that SNPs are distributed among different *Piper* species in a highly uneven manner. A correlation heatmap was used to visualize SNP associations and to construct an auxiliary phylogenetic parsing strategy. We found that species closer in evolutionary distance tend to have fewer SNPs; for example, less than 200 SNPs were identified among the LSC regions of *P. austrosinese*, *P. hainanense*, and *P. nigrum* (Supplementary Table S3). Conversely, species with larger evolutionary distances harbored more than 2000 SNPs in the LSC region, while species with more intermediate distances had 400–700 SNPs. The number of indels can also strongly reflect evolutionary distance, and hence also provides an auxiliary means for assessing the phylogenetic relationships between species. However, the impact of indels on phylogenetic and evolutionary relationships has been a controversial topic (OgdenRosenberg, 2007; Dessimoz Gil, 2010; Nagy et al., 2012; Wambugu et al., 2015). Our study does not provide effective evidence to support whether indels should be considered when reconstructing a phylogenetic tree; for example, we found indels in coding sequences to have little relation to phylogenetic relationships, while those in many other regions, such the SSC, may be detrimental to the determination of phylogenetic relationships. Nevertheless, indel number can to a large extent help in evaluating some hard-to-distinguish phylogenetic relationships.

Overall, our analyses of structural variations determined three species (*P. austrosinese*, *P. hainanense*, and *P. nigrum*) to be closely related to each other, which helped us to exclude conflicting markers that separated those three species during the marker screening process. In addition, we found that *P. umbellatum* should be placed outmost relative to other *Piper* species, since it harbors the most sequence differences. Finally, the other species examined are neither too far nor too close in genetic relationship, and have neither too much nor too little structural variation between them. Therefore, through the integration of many auxiliary conditions, accurate phylogenetic relationships can be obtained with strong evidence. Most of all, for some species in which it is difficult to judge evolutionary distance or some sequences with unusual variations, a more comprehensive analysis of genomic characteristics such as that conducted here will greatly improve the accuracy of determinations.

Markers can be screened among highly variable regions in the chloroplast genome using sliding window and mVista analysis (Cai et al., 2006). Our study showed that direct screening of marker sequences in highly variable sequence intervals, such as the SSC, is also susceptible to difficulty in distinguishing some genetic relationships, which might be caused by the sequences are evolving rapidly in those regions. For example, due to the significant variance in the SSC region in this study, direct reconstruction of the phylogenetic tree from sequences with high Pi values resulted in an incorrect division of the nine species into two evolutionary sets. Therefore, when considering all nine species together, we cannot identify appropriate marker sequences in the SSC, and the phylogenetic results cannot be improved even through using software like Gblock to trim the sequences (Castresana, 2000). Protein-coding genes are generally more conserved and have a more stable sequence structure, especially in their coding sequences (CDSs) (Daniell et al., 2016). Therefore, we focused on evaluating the effectiveness of phylogenetic reconstruction based on CDSs. A total of 12 coding genes were obtained that are highly suitable for phylogenetic reconstruction, which included four genes located in the SSC region. Obviously, CDSs in the SSC region were not significantly affected by high sequence variations; indeed, only a small number of indels were identified in such CDSs (Table 1). Alternatively, it can be said that coding sequences have higher selection pressure and are not prone to variation, while non-coding sequences have lower selection pressure and are continually evolving. In addition, all ideal genes identified here shared some characteristics, such as having a CDS of more than 1,000 bp in length and harboring more than 30 SNPs. It is likely that given SNPs being generated at a certain frequency, enough can be accumulated in a length of sequence so as to accurately reflect evolutionary relationships. Another striking feature of the ideal genes is that all are located on or near the expected curve in the ENC-GC3 plot, which indicates that the codon bias of these genes is weak. In summary, some CDSs with low codon bias are good choices for the reconstruction of *Piper* chloroplast phylogenetic relationships, not only avoiding the adverse effects of abnormal sequence variation that occurs in the SSC region outside CDSs but also providing enough effective SNPs for accurate phylogenetic reconstruction.

## Conclusion

Our study carried out a comparative analysis of the chloroplast genomes of several poorly studied *Piper* species and provided a new strategy for determining previously indistinguishable phylogenetic relationships and key markers in *Piper* chloroplast. Chloroplast genome evolution is usually represented by structural variations such as SSRs, tandem repeats, SNPs, and indels, which constitute important references for measuring the evolutionary relationships among species. Comprehensive consideration of aspects of structural variation (such as the distributions of SNPs, indels, and SSRs) can assist in judging the phylogenetic relationships and correct some classification errors caused by abnormal variations. In addition, because CDS sequences are relatively stable and not affected by high sequence variation, CDS regions are suitable as markers. Those satisfying the marker condition tend to have lower codon preference, be of a certain sequence length, and harbor enough SNPs. All told, this study provides a molecular basis for the phylogenetic classification of *Piper*, analyzes some high genomic variations, and provides effective solutions for analysis of difficult species relationships.

## Data Availability

The assemblies of chloroplast genomes in this research are available in the NCBI GenBank database with the accession number OM717256-OM717263.

## Supplementary material

SUPPLEMENTARY FIGURE S1 The gene map of eight *Piper* chloroplast genomes.

SUPPLEMENTARY FIGURE S2 The bar chart of the number of nucleotide substitutions.

SUPPLEMENTARY FIGURE S3 The phylogenetic tree obtained from the sequences of different regions.

SUPPLEMENTARY TABLE S1 The basic information of each chloroplast genomes.

SUPPLEMENTARY TABLE S2 The information of SSR in eight *Piper* species.

SUPPLEMENTARY TABLE S3 The information of SNP between species.

SUPPLEMENTARY TABLE S4 The information of indels between species.

SUPPLEMENTARY TABLE S5 The Pi distribution for each region.

## References

[B1] Alwadani K. G. Janes J. K. Andrew R. L. (2019). Chloroplast genome analysis of box-ironbark *Eucalyptus* . Mol. Phylogenet. Evol. 136, 76–86. 10.1016/j.ympev.2019.04.001 30954587

[B2] Asmarayani R. (2018). Phylogenetic relationships in malesian–pacific *piper* (Piperaceae) and their implications for systematics. TAXON 67, 693–724. 10.12705/674.2

[B3] Beier S. Thiel T. Münch T. Scholz U. Mascher M. (2017). MISA-Web: A web server for microsatellite prediction. Bioinformatics 33 (16), 2583–2585. 10.1093/bioinformatics/btx198 28398459 PMC5870701

[B4] Benson G. (1999). Tandem repeats finder: A program to analyze DNA sequences. Nucleic Acids Res. 27 (2), 573–580. 10.1093/nar/27.2.573 9862982 PMC148217

[B5] Berndt R. (2013). Revision of the rust genus uromyces on cucurbitaceae. Mycologia 105 (3), 760–780. 10.3852/12-233 23233515

[B6] Cai Z. Penaflor C. Kuehl J. V. Leebens-Mack J. Carlson J. E. dePamphilis C. W. (2006). Complete plastid genome sequences of *drimys, liriodendron*, and *piper*: Implications for the phylogenetic relationships of magnoliids. BMC Evol. Biol. 6, 77. 10.1186/1471-2148-6-77 17020608 PMC1626487

[B7] Castresana J. (2000). Selection of conserved blocks from multiple alignments for their use in phylogenetic analysis. Mol. Biol. Evol. 17 (4), 540–552. 10.1093/oxfordjournals.molbev.a026334 10742046

[B8] Crane P. Friis E. Pedersen K. (1995). The origin and early diversification of angiosperms. Nature 374, 27–33. 10.1038/374027a0

[B9] Daniell H. Lin C. S. Yu M. Chang W. J. (2016). Chloroplast genomes: Diversity, evolution, and applications in genetic engineering. Genome Biol. 17 (1), 134. 10.1186/s13059-016-1004-2 27339192 PMC4918201

[B10] de Vere N. Rich T. C. Trinder S. A. Long C. (2015). DNA barcoding for plants. Methods Mol. Biol. 1245, 101–118. 10.1007/978-1-4939-1966-6_8 25373752

[B11] de Vries J. Archibald J. M. (2018). Plastid genomes. Curr. Biol. 28 (8), R336–R337. 10.1016/j.cub.2018.01.027 29689202

[B12] Dessimoz C. Gil M. (2010). Phylogenetic assessment of alignments reveals neglected tree signal in gaps. Genome Biol. 11, R37. 10.1186/gb-2010-11-4-r37 20370897 PMC2884540

[B13] Fan Y. Jin Y. Ding M. Tang Y. Cheng J. Zhang K. (2021). The complete chloroplast genome sequences of eight fagopyrum species: Insights into genome evolution and phylogenetic relationships. Front. Plant Sci. 12, 799904. 10.3389/fpls.2021.799904 34975990 PMC8715082

[B14] Favre A. Pringle J. S. Heckenhauer J. Kozuharova E. Gao Q. Lemmon E. M. (2020). Phylogenetic relationships and sectional delineation within *Gentiana* (Gentianaceae). TAXON 69, 1221–1238. 10.1002/tax.12405

[B15] Frenzke L. Scheiris E. Pino G. Symmank L. Goetghebeur P. Neinhuis C. (2015). A revised infrageneric classification of the genus *Peperomia* (Piperaceae). TAXON 64, 424–444. 10.12705/643.4

[B16] Gao X. Zhang X. Meng H. Li J. Zhang D. Liu C. (2018). Comparative chloroplast genomes of paris sect. Marmorata: Insights into repeat regions and evolutionary implications. BMC Genomics 19 (10), 878. 10.1186/s12864-018-5281-x 30598104 PMC6311911

[B17] Gaudeul M. Giraud T. Kiss L. Shykoff J. A. (2011). Nuclear and chloroplast microsatellites show multiple introductions in the worldwide invasion history of common ragweed, *Ambrosia artemisiifolia* . PLoS One 6 (3), e17658. 10.1371/journal.pone.0017658 21423697 PMC3053376

[B18] Group T. A. P. (2016). An update of the angiosperm phylogeny group classification for the orders and families of flowering plants: Apg IV. Bot. J. Linn. Soc. 181, 1–20. 10.1111/boj.12385

[B19] Hansen D. R. Dastidar S. G. Cai Z. Penaflor C. Kuehl J. V. Boore J. L. (2007). Phylogenetic and evolutionary implications of complete chloroplast genome sequences of four early-diverging angiosperms: *Buxus* (Buxaceae), *Chloranthus* (Chloranthaceae), *Dioscorea* (Dioscoreaceae), and *Illicium* (Schisandraceae). Mol. Phylogenet. Evol. 45 (2), 547–563. 10.1016/j.ympev.2007.06.004 17644003

[B20] Huang B. Ruess H. Liang Q. Colleoni C. Spooner D. M. (2019). Analyses of 202 plastid genomes elucidate the phylogeny of *Solanum* section Petota. Sci. Rep. 9 (1), 4454. 10.1038/s41598-019-40790-5 30872631 PMC6418237

[B21] Jansen R. K. Cai Z. Raubeson L. A. Daniell H. Depamphilis C. W. Leebens-Mack J. (2007). Analysis of 81 genes from 64 plastid genomes resolves relationships in angiosperms and identifies genome-scale evolutionary patterns. Proc. Natl. Acad. Sci. U. S. A. 104 (49), 19369–19374. 10.1073/pnas.0709121104 18048330 PMC2148296

[B22] Jaramillo A. Callejas R. Davidson C. Smith J. F. Stevens A. C. Tepe E. J. (2008). A phylogeny of the tropical genus *piper* using ITS and the chloroplast intron psbJ-petA. Syst. Bot. 33, 647–660. 10.1600/036364408786500244

[B23] Jiao Y. Jia H. M. Li X. W. Chai M. L. Jia H. J. Chen Z. (2012). Development of simple sequence repeat (SSR) markers from a genome survey of Chinese bayberry (*Myrica rubra*). BMC Genomics 13, 201. 10.1186/1471-2164-13-201 22621340 PMC3505174

[B24] Jin D. P. Choi I. S. Choi B. H. (2019). Plastid genome evolution in tribe desmodieae (fabaceae: Papilionoideae). PLoS One 14 (6), e0218743. 10.1371/journal.pone.0218743 31233545 PMC6590825

[B25] Jin J. J. Yu W. B. Yang J. B. Song Y. dePamphilis C. W. Yi T. S. (2020). GetOrganelle: A fast and versatile toolkit for accurate de novo assembly of organelle genomes. Genome Biol. 21 (1), 241. 10.1186/s13059-020-02154-5 32912315 PMC7488116

[B26] Kassambara A. (2019). ggcorrplot: Visualization of a Correlation Matrix using 'ggplot2'. Available at: https://cran.r-project.org/web/packages/ggthemes/index.html

[B27] Katoh K. Standley D. M. (2013). MAFFT multiple sequence alignment software version 7: Improvements in performance and usability. Mol. Biol. Evol. 30 (4), 772–780. 10.1093/molbev/mst010 23329690 PMC3603318

[B28] Kearse M. Moir R. Wilson A. Stones-Havas S. Cheung M. Sturrock S. (2012). Geneious basic: An integrated and extendable desktop software platform for the organization and analysis of sequence data. Bioinformatics 28 (12), 1647–1649. 10.1093/bioinformatics/bts199 22543367 PMC3371832

[B29] Kurtz S. Choudhuri J. V. Ohlebusch E. SChleiermaCher C. Stoye J. GiegeRich R. (2001). REPuter: The manifold applications of repeat analysis on a genomic scale. Nucleic Acids Res. 29 (22), 4633–4642. 10.1093/nar/29.22.4633 11713313 PMC92531

[B30] Liao M. Gao X. F. Zhang J. Y. Deng H. N. Xu B. (2021). Comparative chloroplast genomics of *Sophora* species: Evolution and phylogenetic relationships in the early-diverging legume subfamily papilionoideae (fabaceae). Front. Plant Sci. 12, 778933. 10.3389/fpls.2021.778933 34975964 PMC8716937

[B31] Lim J. Y. Marshall C. R. Zimmer E. A. Wagner W. L. (2019). Multiple colonizations of the Pacific by *Peperomia* (Piperaceae): Complex patterns of long-distance dispersal and parallel radiations on the Hawaiian Islands. J. Biogeogr. 46, 2651–2662. 10.1111/jbi.13717

[B32] Liu Y. Liang Y. M. Ono Y. (2020). Taxonomic revision of species of Kuehneola and Phragmidium on Rosa, including two new species from China. Mycologia 112 (4), 742–752. 10.1080/00275514.2020.1753426 32469695

[B33] Liu Z.-F. Ma H. Ci X.-Q. Li L. Song Y. Liu B. (2021). Can plastid genome sequencing be used for species identification in Lauraceae? Bot. J. Linn. Soc. 197, 1–14. 10.1093/botlinnean/boab018

[B34] Lowe T. M. Eddy S. R. (1997). tRNAscan-SE: A program for improved detection of transfer RNA genes in genomic sequence. Nucleic Acids Res. 25 (5), 955–964. 10.1093/nar/25.5.955 9023104 PMC146525

[B35] Ma C. Y. (1990). Review of the classification system on the genus Sophora. Acta. Phytotaxon. Sin. 10, 77–86.

[B36] Magallón S. Castillo A. (2009). Angiosperm diversification through time. Am. J. Bot. 96 (1), 349–365. 10.3732/ajb.0800060 21628193

[B37] Marie-France D. Marie-Hélène P. Rémy J. P. (2004). Use of chloroplast microsatellites to differentiate oak populations. Ann. For. Sci. 61, 825–830. 10.1051/forest:2004078

[B38] Nagy L. G. Kocsubé S. Csanádi Z. Kovacs G. M. Petkovits T. Vagvolgyi C. (2012). Re-mind the gap! Insertion - deletion data reveal neglected phylogenetic potential of the nuclear ribosomal internal transcribed spacer (ITS) of fungi. PloS One 7, e49794. 10.1371/journal.pone.0049794 23185439 PMC3501463

[B39] Nick P. (2020). Tracking footprints of plastid evolution. Protoplasma 257 (4), 1019–1020. 10.1007/s00709-020-01526-9 32572583 PMC7329753

[B40] Ogden T. H. Rosenberg M. S. (2007). How should gaps be treated in parsimony? A comparison of approaches using simulation. Mol. Phylogenet. Evol. 42, 817–826. 10.1016/j.ympev.2006.07.021 17011794

[B41] Pahlich E. Gerlitz C. (1980). Deviations from michaelis-menten behaviour of plant glutamate dehydrogenase with ammonium as variable substrate. Phytochemistry 19, 11–13. 10.1016/0031-9422(80)85004-7

[B42] Patel R. K. Jain M. (2012). NGS QC toolkit: A toolkit for quality control of next generation sequencing data. PLoS One 7 (2), e30619. 10.1371/journal.pone.0030619 22312429 PMC3270013

[B43] Peden J. F. (1999). Analysis of codon usage. PhD thesis. UK: University of Nottingham.

[B44] Piya S. Nepal M. P. (2013). Characterization of nuclear and chloroplast microsatellite markers for *Falcaria vulgaris* (apiaceae). Am. J. Plant Sci. 4 (3), 590–595. 10.4236/ajps.2013.43077

[B45] Rambaut A. (1996). Se-Al: Sequence alignment editor. version 2.0. http://tree.bio.ed.ac.uk/software/seal/ .

[B46] Ronquist F. Teslenko M. van der Mark P. Ayres D. L. Darling A. Hohna S. (2012). MrBayes 3.2: Efficient bayesian phylogenetic inference and model choice across a large model space. Syst. Biol. 61 (3), 539–542. 10.1093/sysbio/sys029 22357727 PMC3329765

[B47] Rozas J. Ferrer-Mata A. Sánchez-DelBarrio J. C. Guirao-Rico S. Librado P. Ramos-Onsins S. E. (2017). DnaSP 6: DNA sequence polymorphism analysis of large data sets. Mol. Biol. Evol. 34 (12), 3299–3302. 10.1093/molbev/msx248 29029172

[B48] Ruhlman T. A. Jansen R. K. (2014). The plastid genomes of flowering plants. Methods Mol. Biol. 1132, 3–38. 10.1007/978-1-62703-995-6_1 24599844

[B49] Ruhlman T. Lee S. B. Jansen R. K. Hostetler J. B. Tallon L. J. Town C. D. (2006). Complete plastid genome sequence of daucus carota: Implications for biotechnology and phylogeny of angiosperms. BMC Genomics 7, 222. 10.1186/1471-2164-7-222 16945140 PMC1579219

[B50] Sauquet H. Magallón S. (2018). Key questions and challenges in angiosperm macroevolution. New Phytol. 219 (4), 1170–1187. 10.1111/nph.15104 29577323

[B51] Sen S. Dayanandan S. Davis T. Ganesan R. Jagadish M. R. Mathew P. J. (2019). Origin and evolution of the genus *piper* in peninsular India. Mol. Phylogenet. Evol. 138, 102–113. 10.1016/j.ympev.2019.05.033 31132521

[B52] Shi L. Chen H. Jiang M. Wang L. Wu X. Huang L. (2019). CPGAVAS2, an integrated plastome sequence annotator and analyzer. Nucleic Acids Res. 47 (W1), W65–W73. 10.1093/nar/gkz345 31066451 PMC6602467

[B53] Simmonds S. E. Smith J. F. Davidson C. Buerki S. (2021). Phylogenetics and comparative plastome genomics of two of the largest genera of angiosperms, *Piper* and *Peperomia* (Piperaceae). Mol. Phylogenet. Evol. 163, 107229. 10.1016/j.ympev.2021.107229 34129936

[B54] Soltis D. E. Bell C. D. Kim S. Soltis P. S. (2008). Origin and early evolution of angiosperms. Ann. N. Y. Acad. Sci. 1133, 3–25. 10.1196/annals.1438.005 18559813

[B55] Soltis P. S. Soltis D. E. (2004). The origin and diversification of angiosperms. Am. J. Bot. 91 (10), 1614–1626. 10.3732/ajb.91.10.1614 21652312

[B56] Song Y. A.-O. X. Chen Y. Lv J. Xu J. Zhu S. Li M. A.-O. (2019). Comparative chloroplast genomes of sorghum species: Sequence divergence and phylogenetic relationships. Biomed. Res. Int. 2019, 5046958. 10.1155/2019/5046958 31016191 PMC6444266

[B57] Stamatakis A. (2014). RAxML version 8: A tool for phylogenetic analysis and post-analysis of large phylogenies. Bioinformatics 30 (9), 1312–1313. 10.1093/bioinformatics/btu033 24451623 PMC3998144

[B58] Tamura K. Stecher G. Kumar S. (2021). MEGA11: Molecular evolutionary genetics analysis version 11. Mol. Biol. Evol. 38 (7), 3022–3027. 10.1093/molbev/msab120 33892491 PMC8233496

[B59] Tillich M. Lehwark P. Pellizzer T. Ulbricht-Jones E. S. Fischer A. Bock R. (2017). GeSeq - versatile and accurate annotation of organelle genomes. Nucleic Acids Res. 45 (W1), W6–W11. 10.1093/nar/gkx391 28486635 PMC5570176

[B60] Tsoong Pu-Chiu M. C.-Y. (1981). A study on the genus Sophora Linn. J. Syst. Evol. 19, 143–167.

[B61] Wambugu P. Brozynska M. Furtado A. Waters D. L. Henry R. J. (2015). Relationships of wild and domesticated rices (Oryza AA genome species) based upon whole chloroplast genome sequences. Sci. Rep. 5, 13957. 10.1038/srep13957 26355750 PMC4564799

[B62] Wickham H. (2016). ggplot2: Elegant graphics for data analysis. New York: Springer-Verlag. Available at: https://ggplot2.tidyverse.org .

[B63] Yang J. B. Li D. Z. Li H. T. (2014). Highly effective sequencing whole chloroplast genomes of angiosperms by nine novel universal primer pairs. Mol. Ecol. Resour. 14 (5), 1024–1031. 10.1111/1755-0998.12251 24620934

