# Supplementary figures and images for: Comparative analysis of the chloroplast genomes of eight Piper species and insights into the utilization of structural variation in phylogenetic analysis
Source: Front Genet. 2022 Sep 29;13:925252. doi: 10.3389/fgene.2022.925252 (PMC9556897; doi:10.3389/fgene.2022.925252)

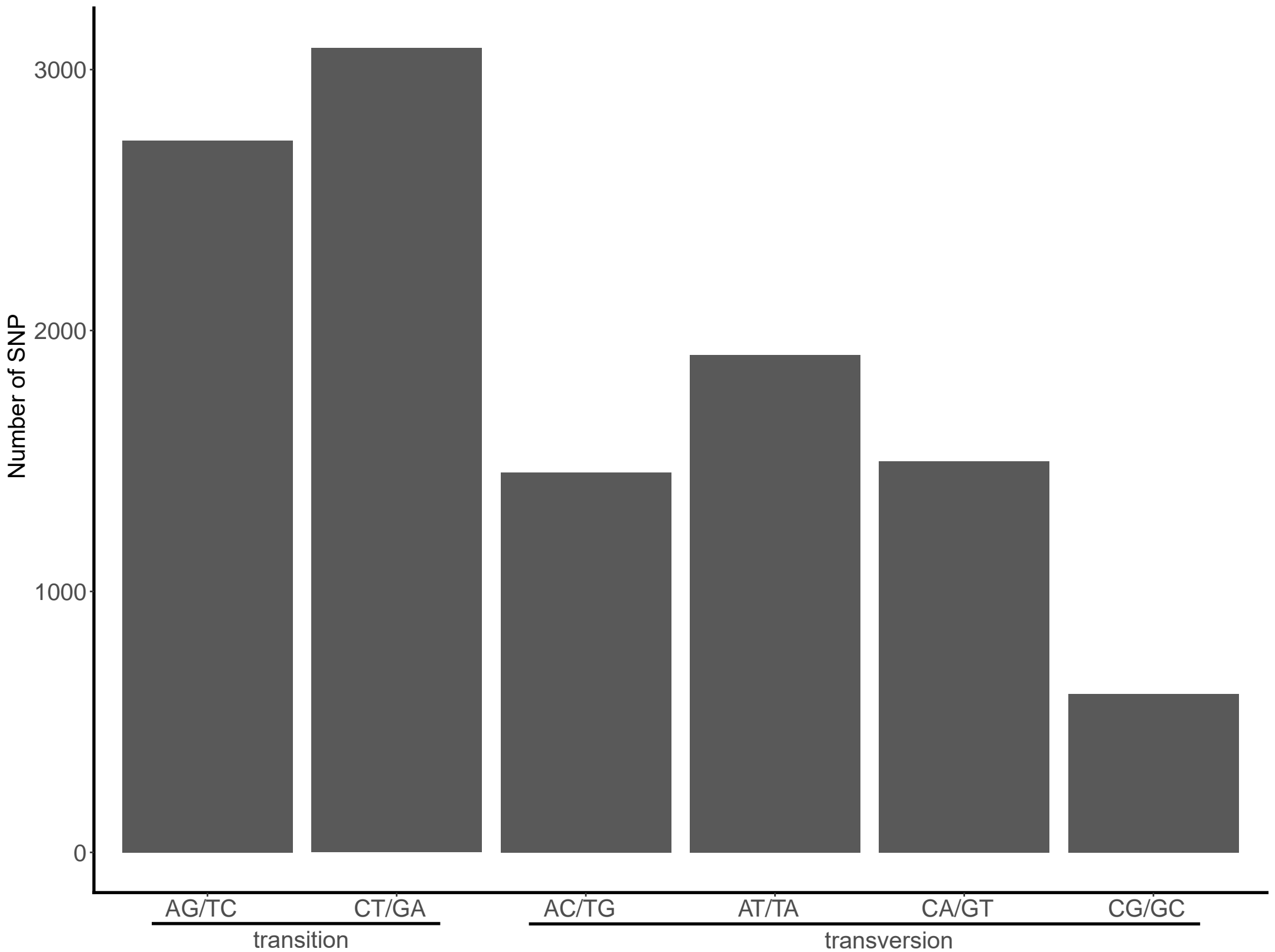

**Figure S2.** The bar chart of the number of nucleotide substitutions.

Supplement: Supplementary file 3 [file Image2.pdf]
