# Supplementary material for: Comparative analysis of the chloroplast genomes of eight Piper species and insights into the utilization of structural variation in phylogenetic analysis
Source: Front Genet. 2022 Sep 29;13:925252. doi: 10.3389/fgene.2022.925252 (PMC9556897; doi:10.3389/fgene.2022.925252)
Supplement: Supplementary file 4 [file Image3.pdf]

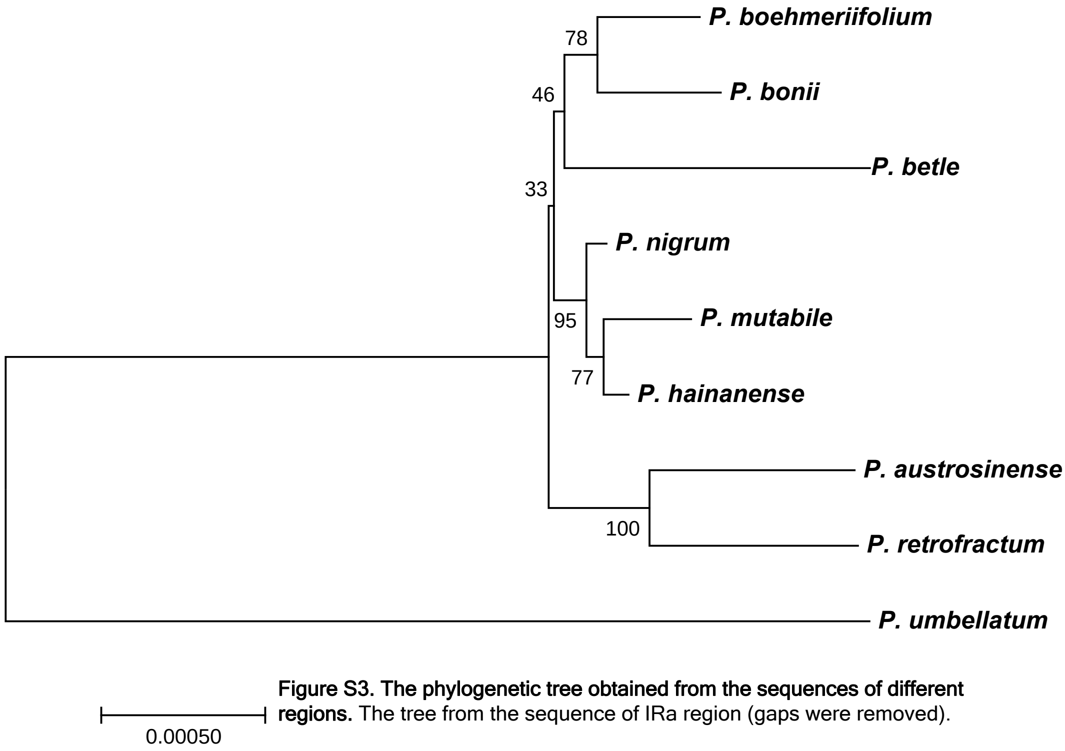

Figure S3. The phylogenetic tree obtained from the sequences of different regions. The tree from the sequence of IRa region (gaps were removed).

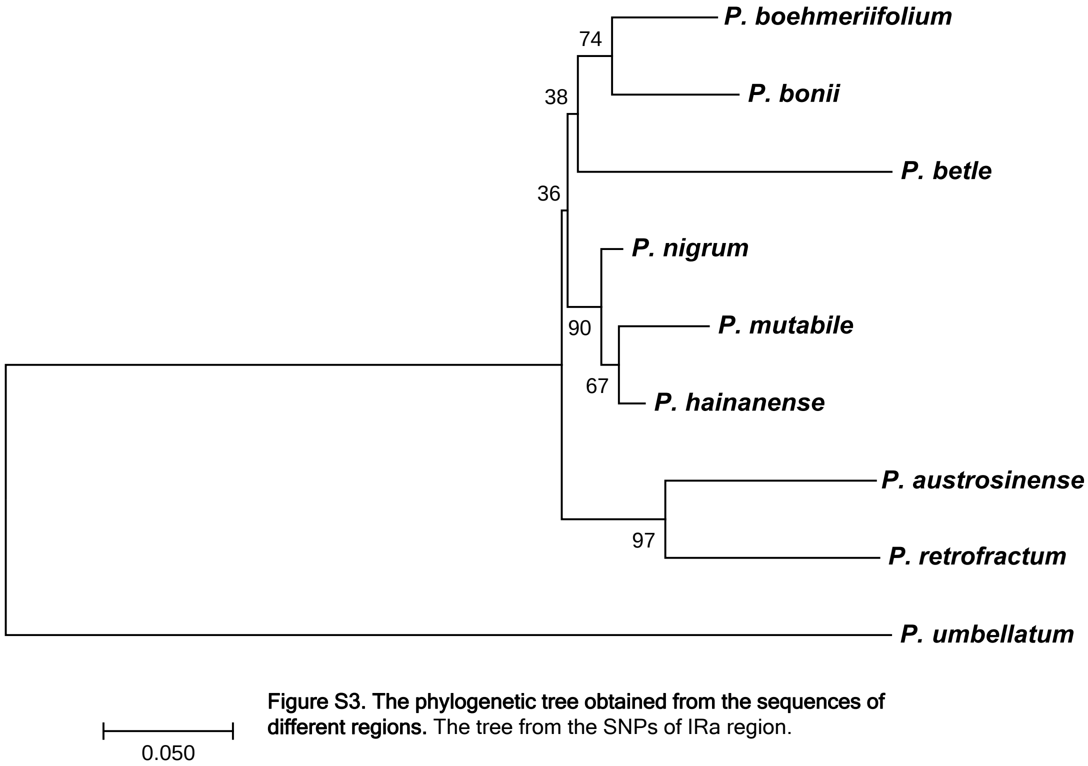

Figure S3. The phylogenetic tree obtained from the sequences of different regions. The tree from the SNPs of IRa region.

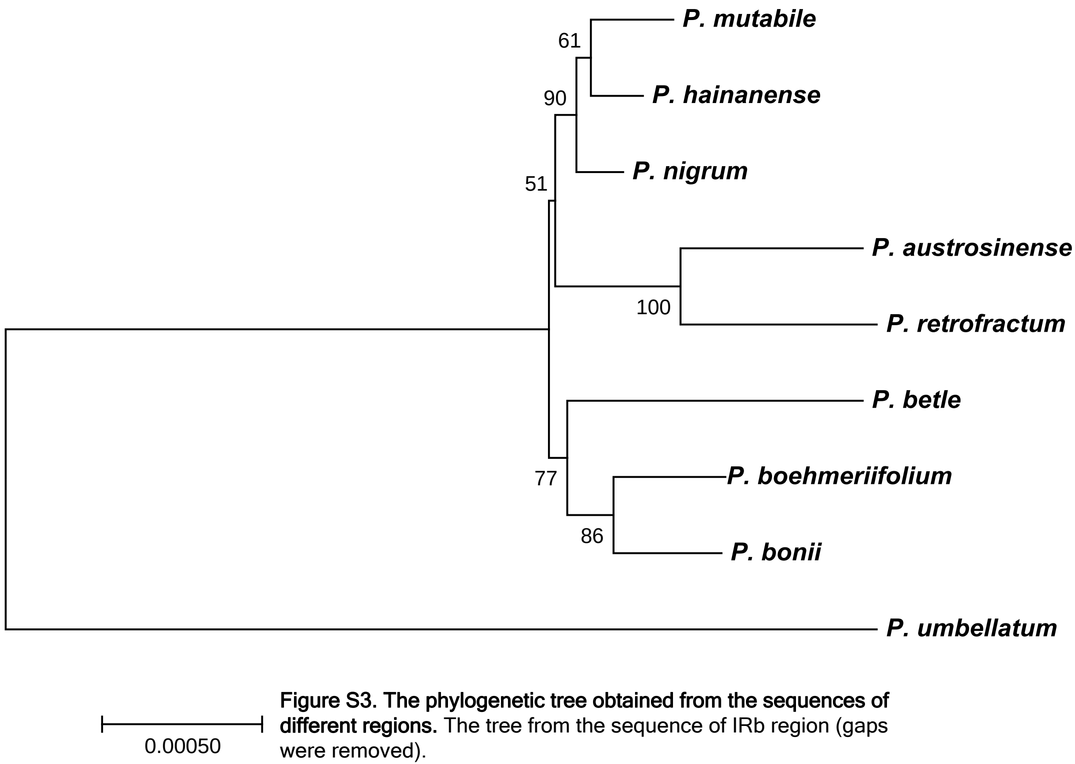

Figure S3. The phylogenetic tree obtained from the sequences of different regions. The tree from the sequence of IRb region (gaps were removed).

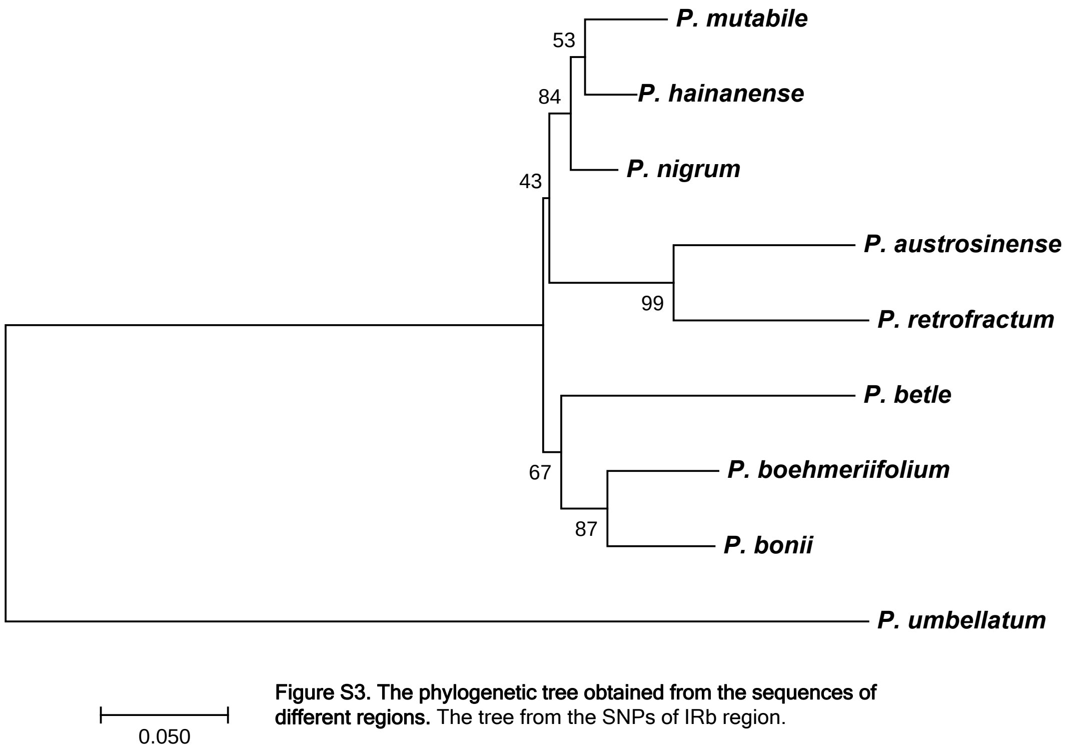

Figure S3. The phylogenetic tree obtained from the sequences of different regions. The tree from the SNPs of IRb region.

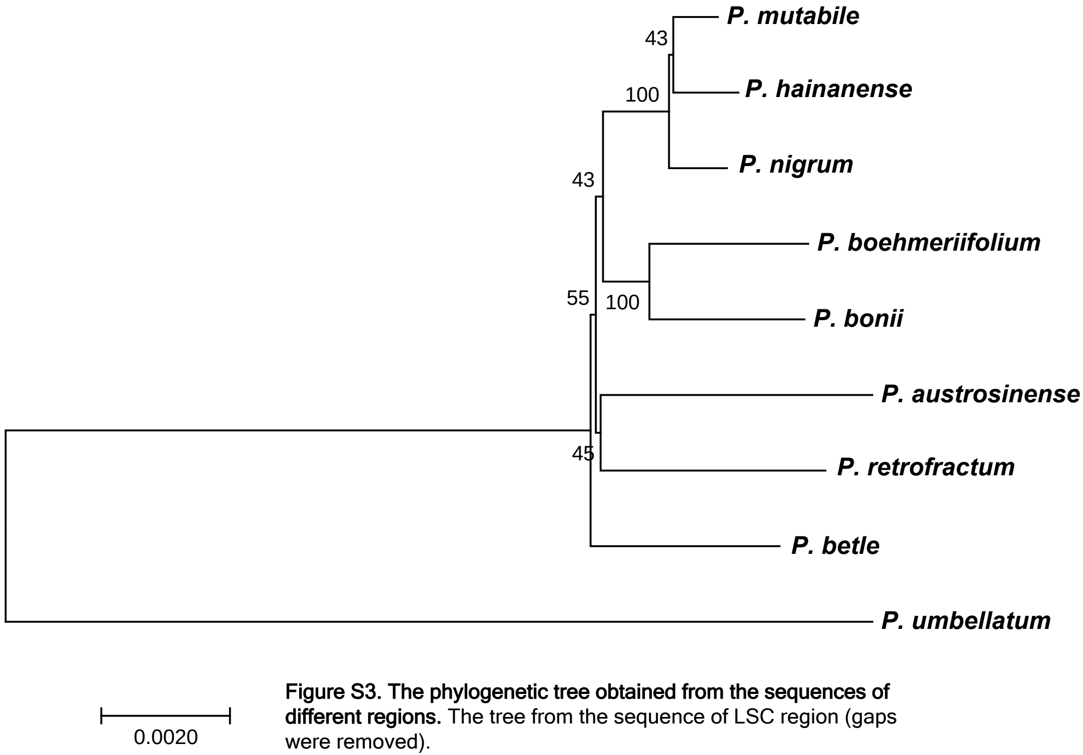

Figure S3. The phylogenetic tree obtained from the sequences of different regions. The tree from the sequence of LSC region (gaps were removed).

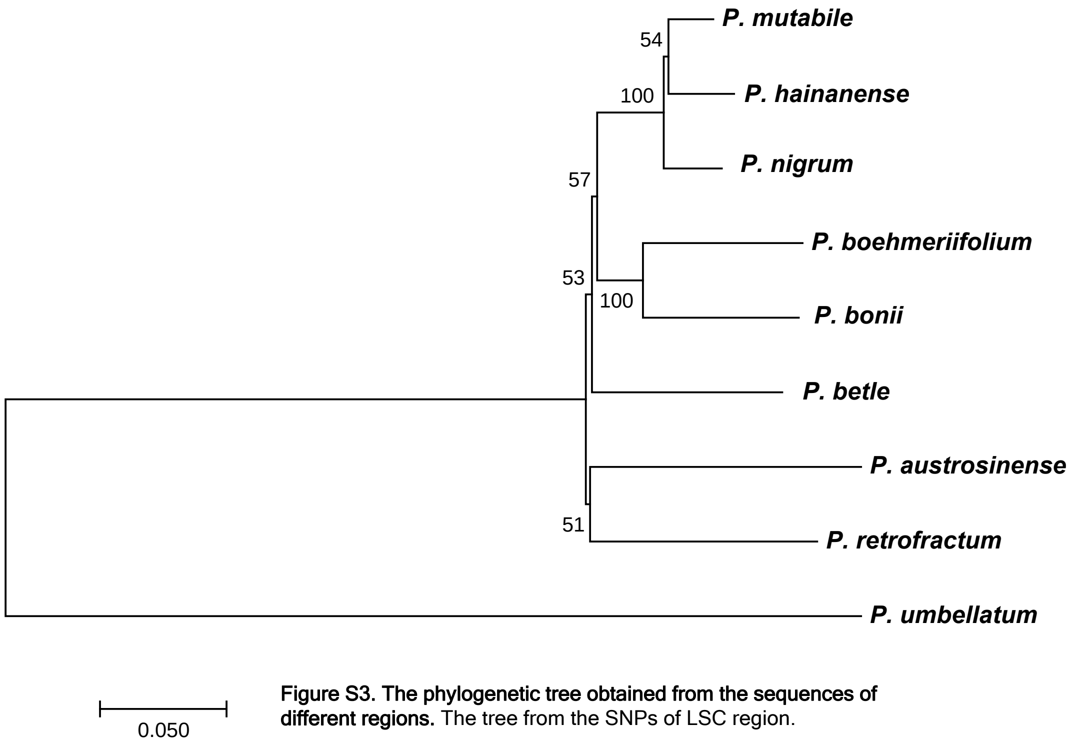

Figure S3. The phylogenetic tree obtained from the sequences of different regions. The tree from the SNPs of LSC region.

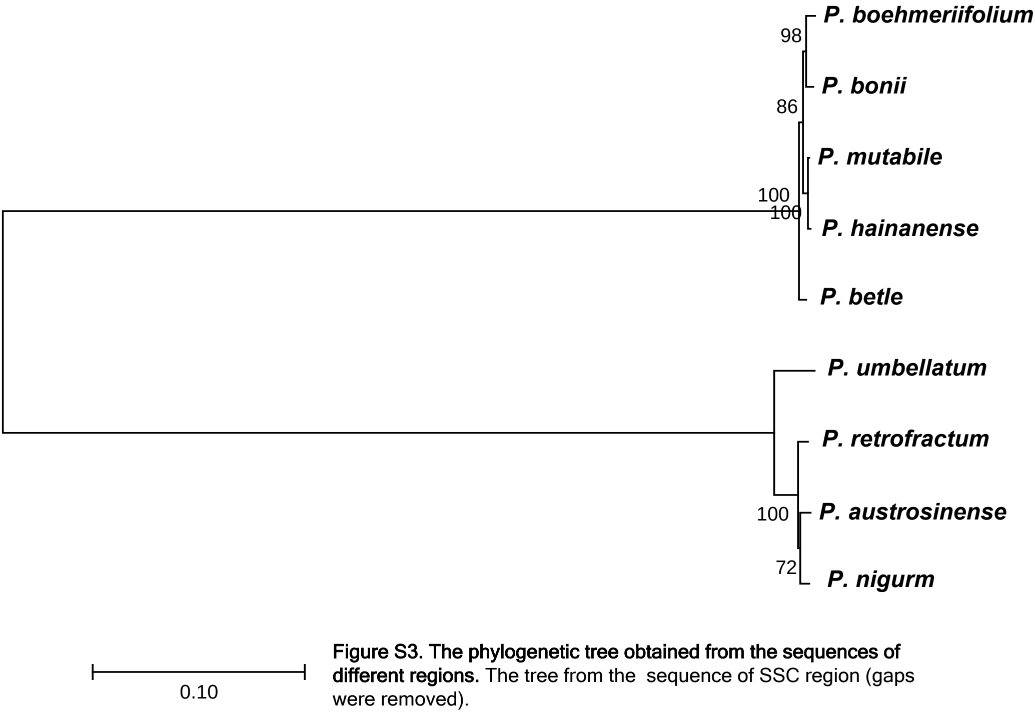

Figure S3. The phylogenetic tree obtained from the sequences of different regions. The tree from the sequence of SSC region (gaps were removed).

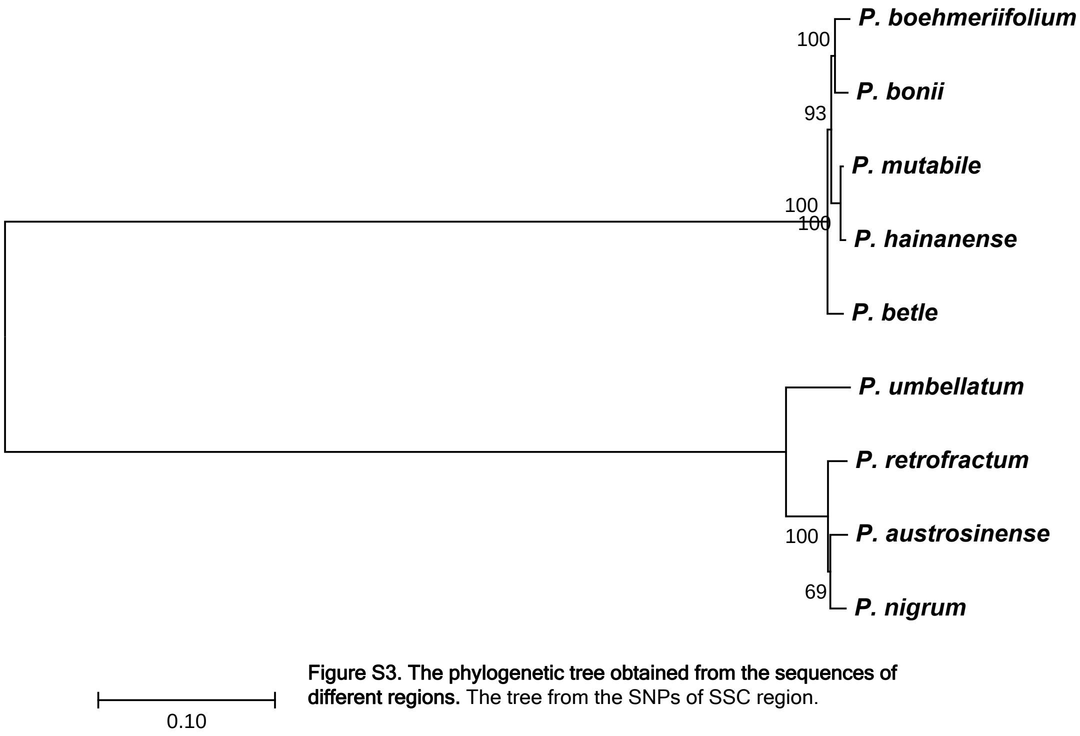

Figure S3. The phylogenetic tree obtained from the sequences of different regions. The tree from the SNPs of SSC region.

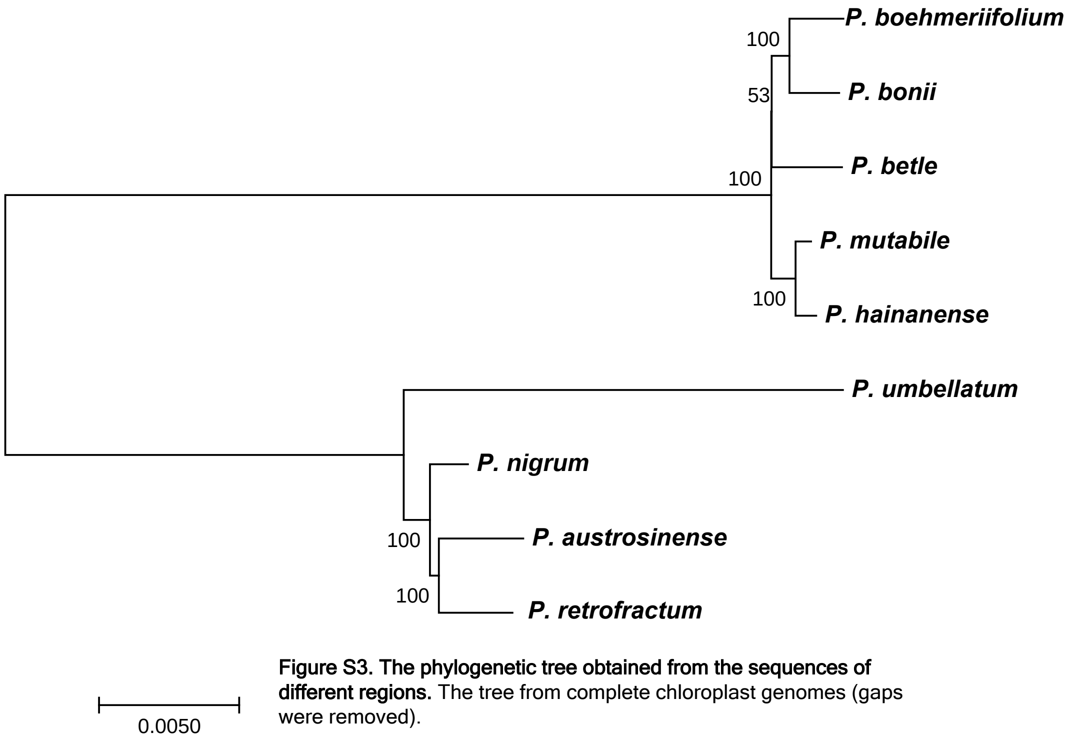

Figure S3. The phylogenetic tree obtained from the sequences of different regions. The tree from complete chloroplast genomes (gaps were removed).

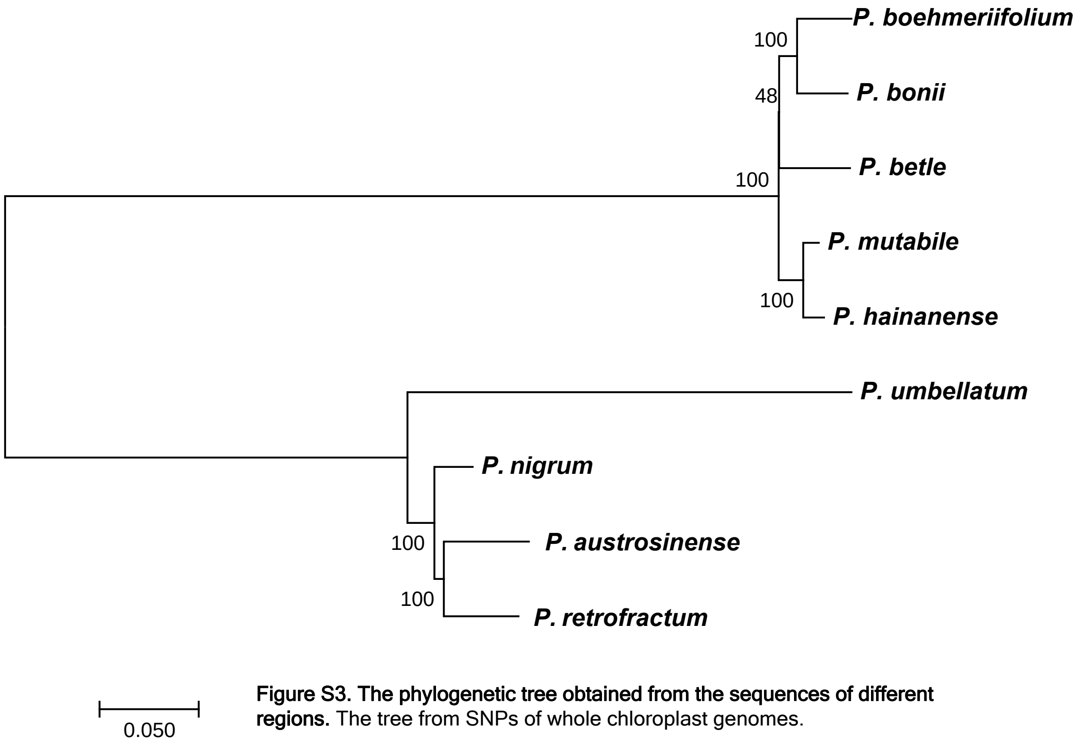

Figure S3. The phylogenetic tree obtained from the sequences of different regions. The tree from SNPs of whole chloroplast genomes.
