# Supplementary material for: Comparative analysis of the chloroplast genomes of eight Piper species and insights into the utilization of structural variation in phylogenetic analysis
Source: Front Genet. 2022 Sep 29;13:925252. doi: 10.3389/fgene.2022.925252 (PMC9556897; doi:10.3389/fgene.2022.925252)
Supplement: Supplementary file 8 [file Image1.pdf]

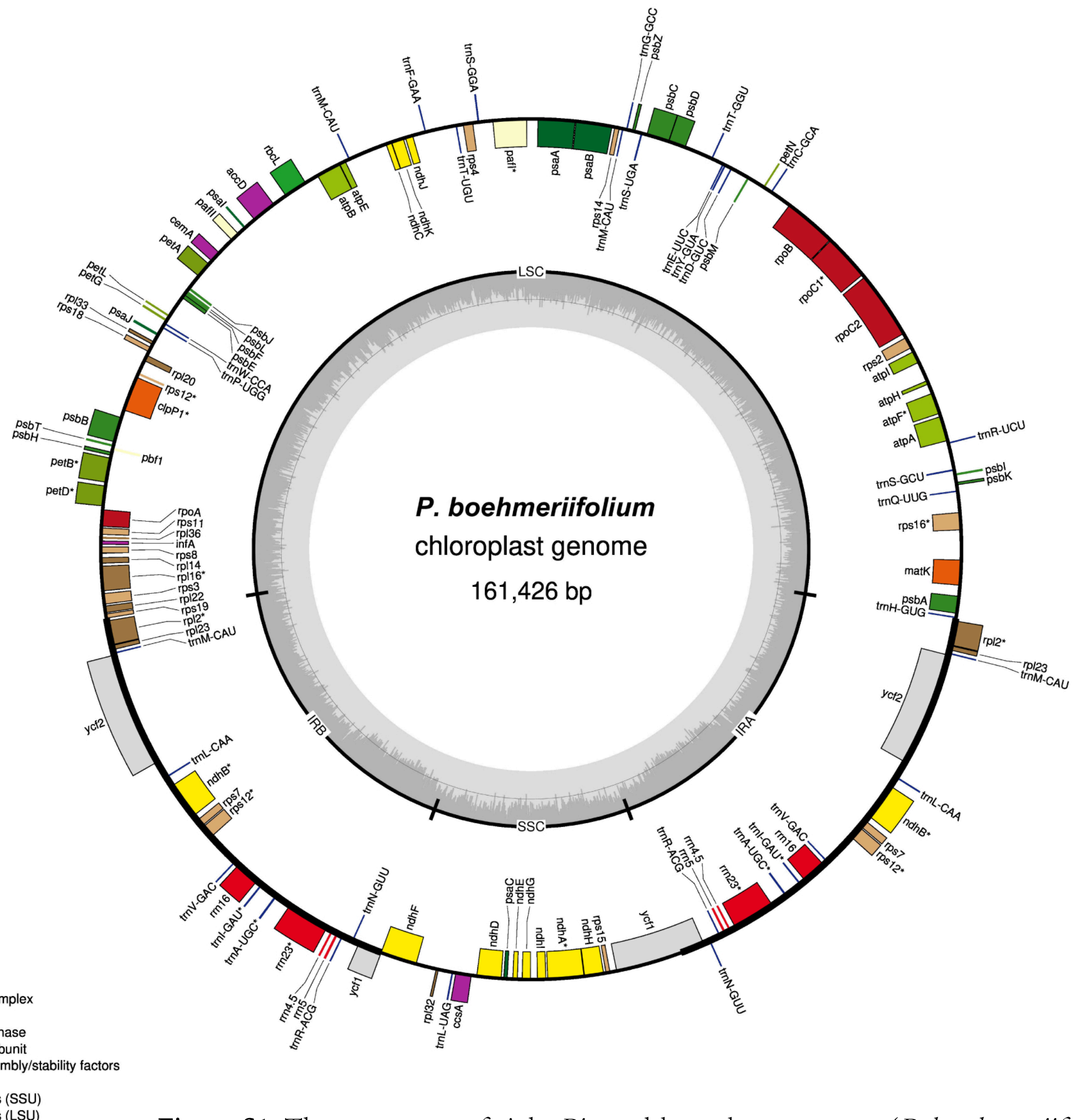

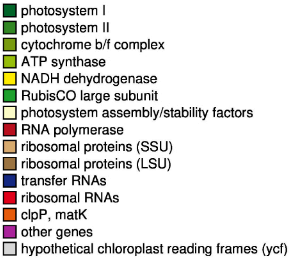

Figure S1. The gene map of eight *Piper* chloroplast genomes (*P. boehmeriifolium*).

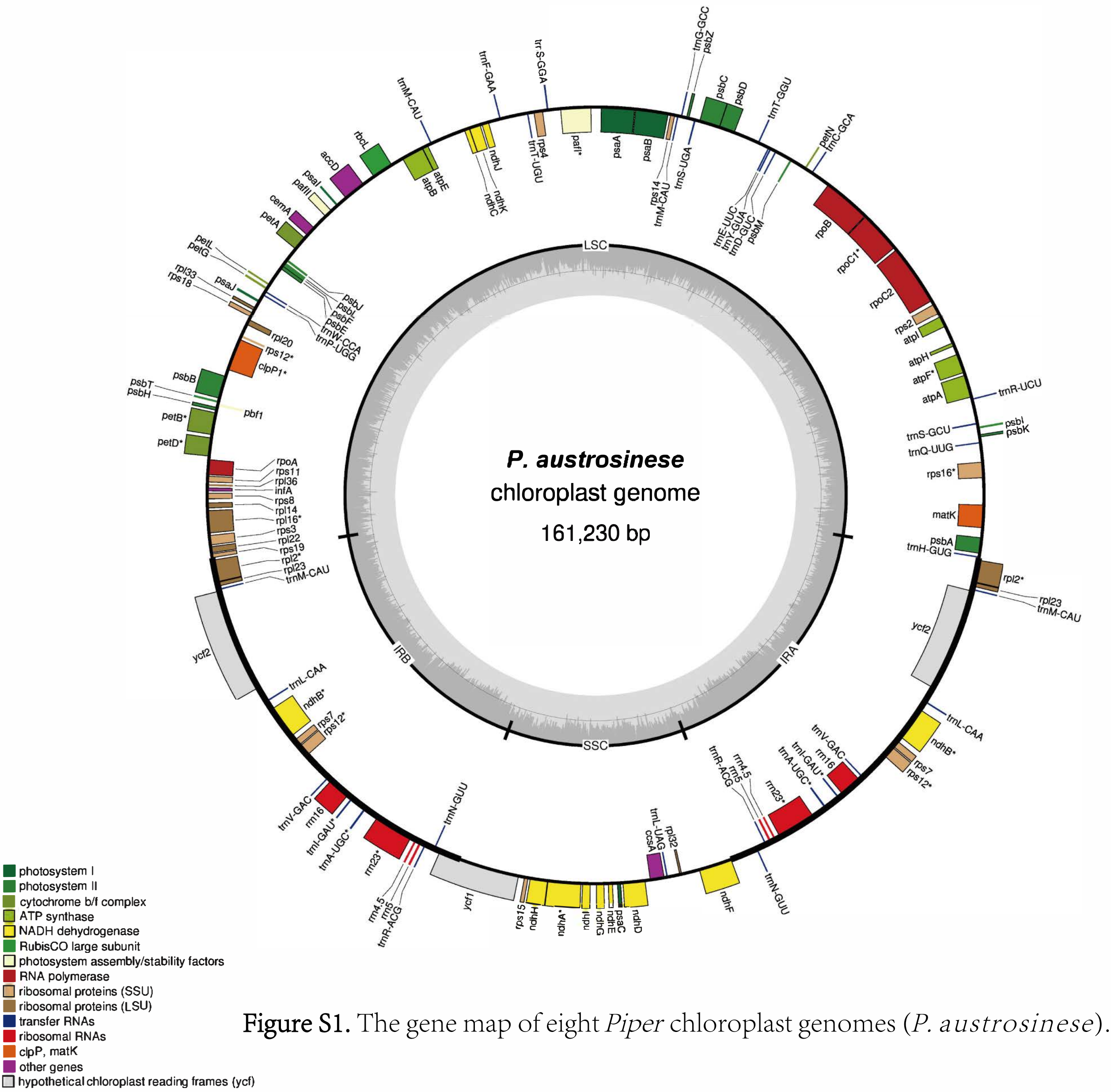

Figure S1. The gene map of eight *Piper* chloroplast genomes (*P. austrosinese*).

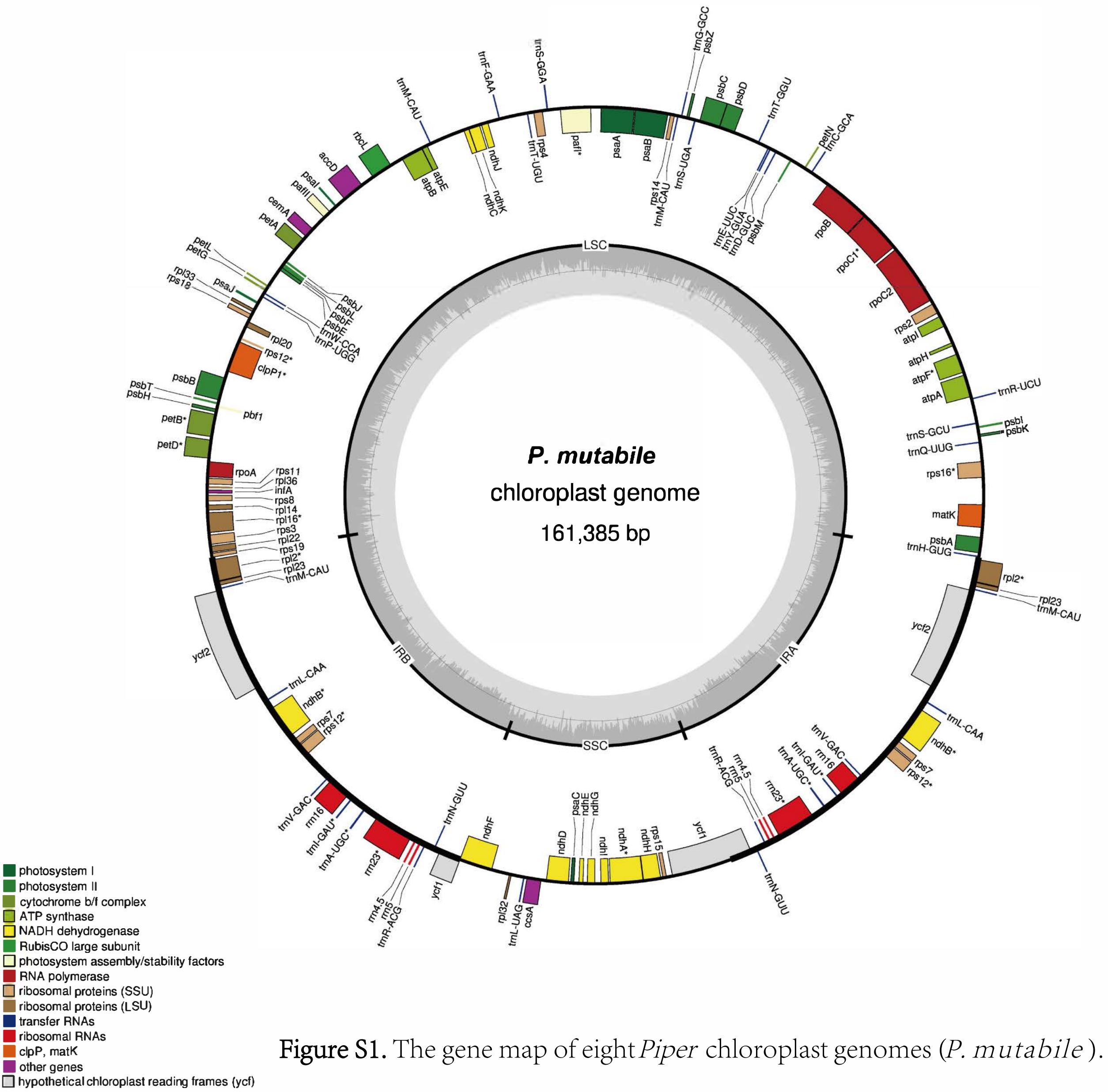

Figure S1. The gene map of eight *Piper* chloroplast genomes (*P. mutabile*).

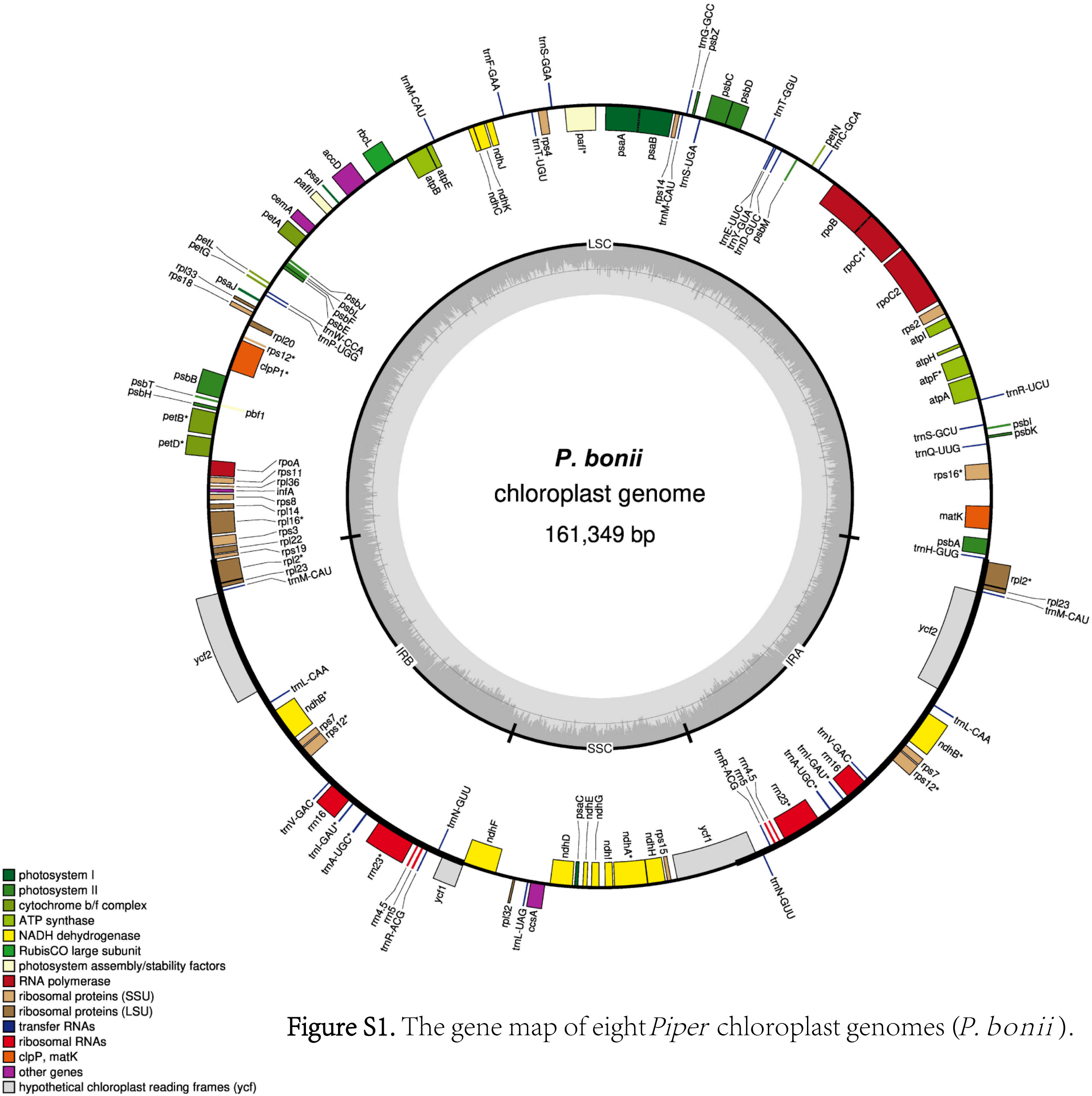

Figure S1. The gene map of eight *Piper* chloroplast genomes (*P. bonii*).

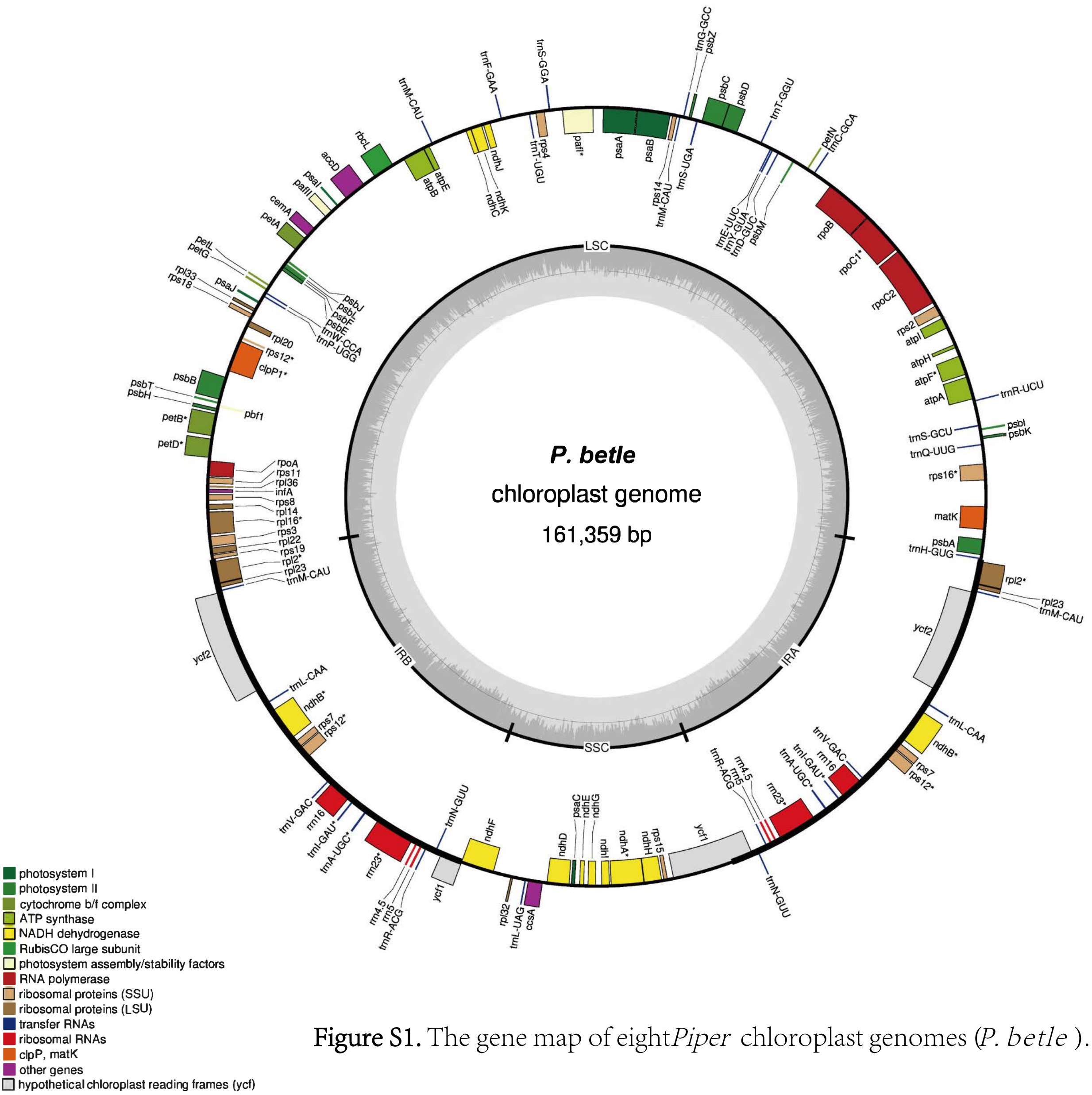

Figure S1. The gene map of eight *Piper* chloroplast genomes (*P. betle*).

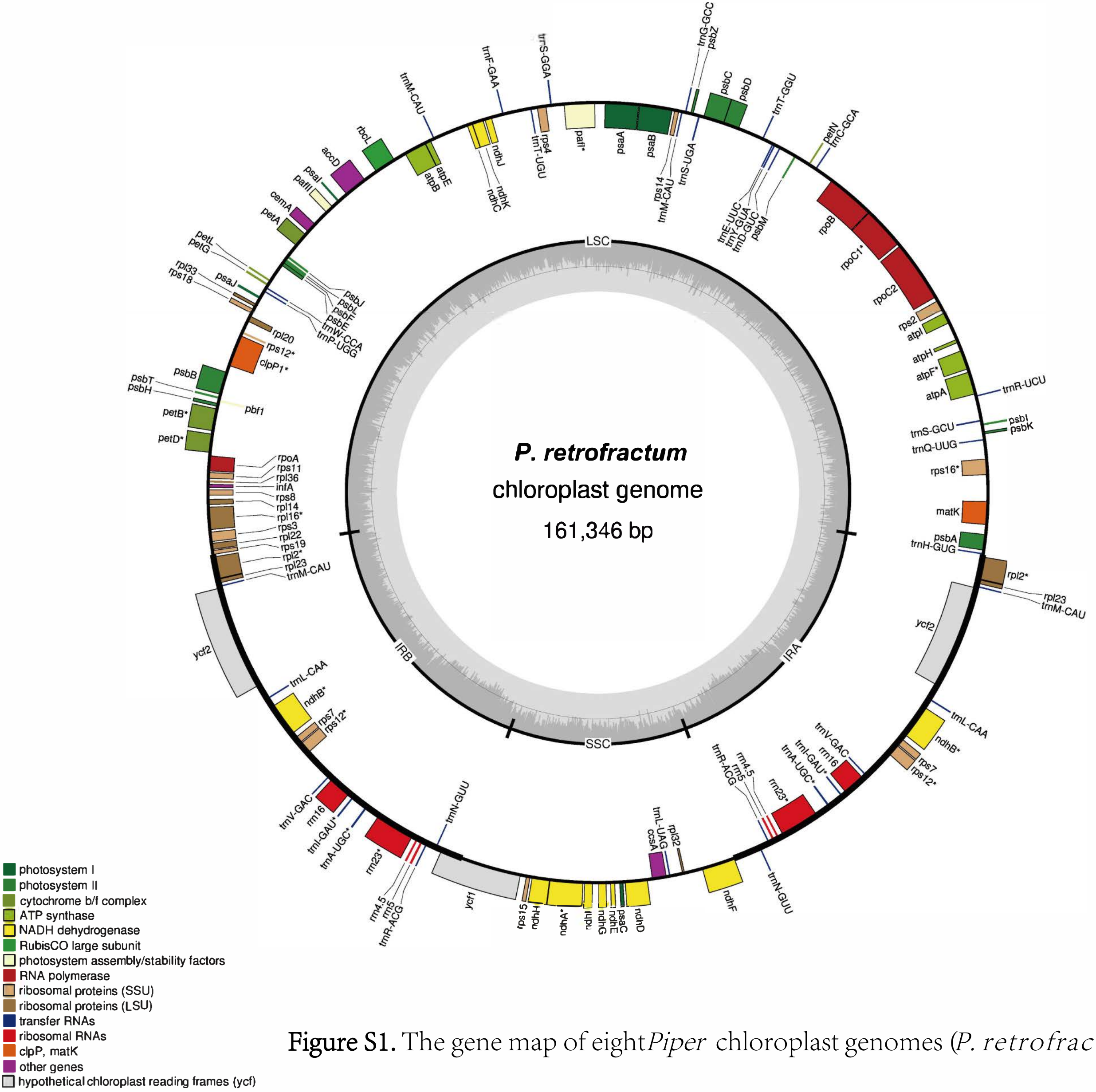

Figure S1. The gene map of eight *Piper* chloroplast genomes (*P. retrofractum*).

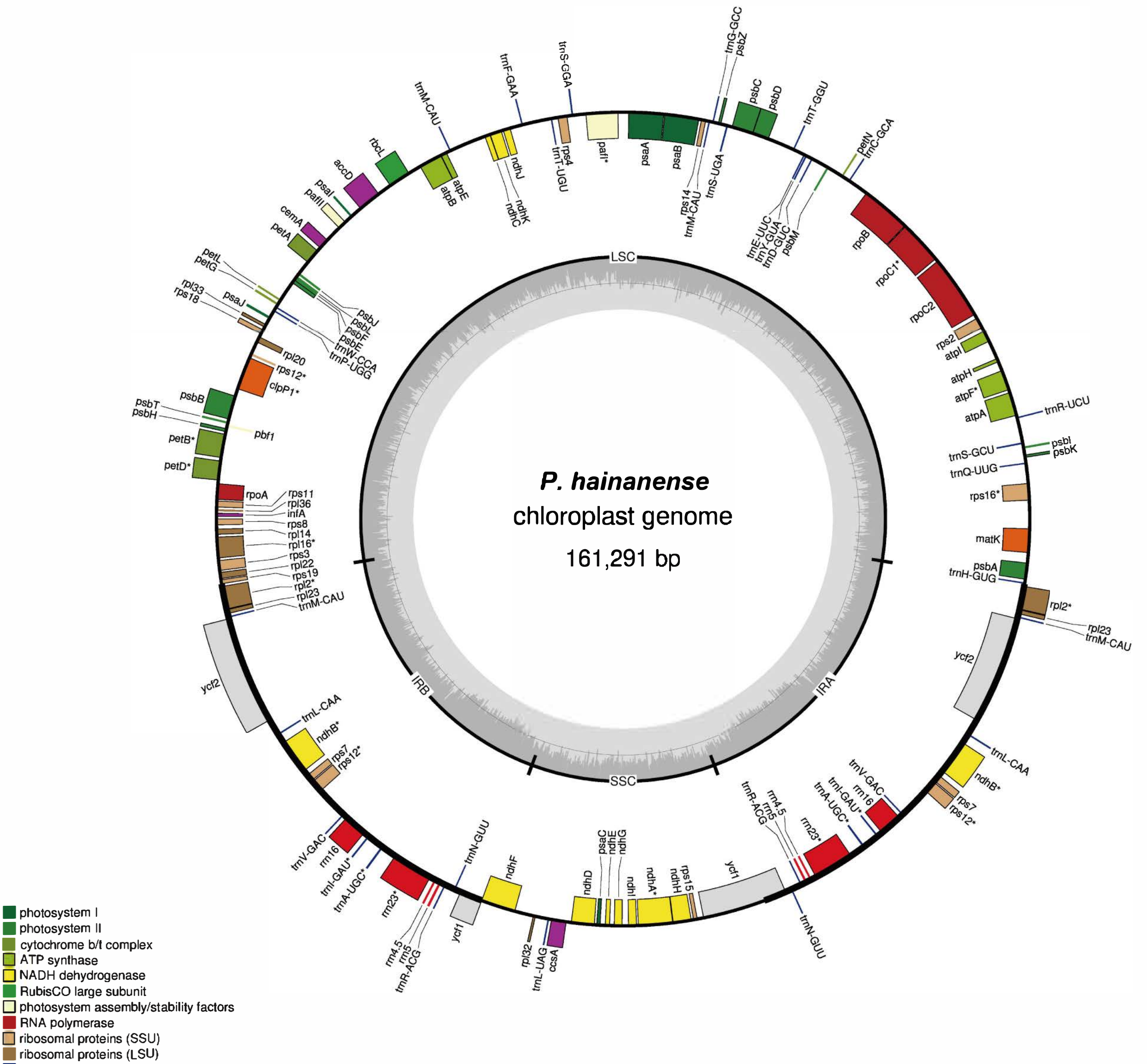

Figure S1. The gene map of eight *Piper* chloroplast genomes (*P. hainanense*).

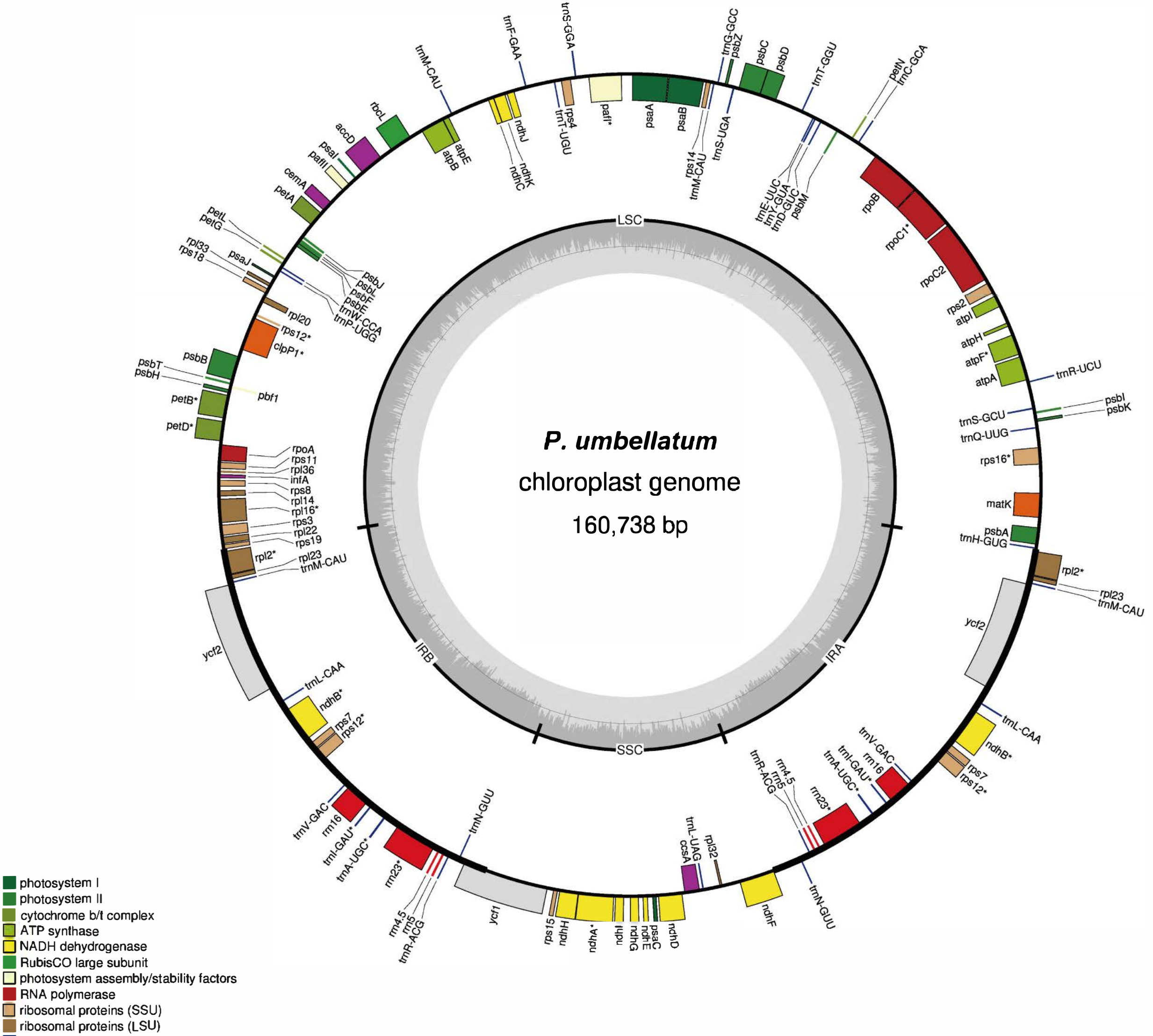

Figure S1. The gene map of eight *Piper* chloroplast genomes (*P. umbellatum*).
